# Evaluating Pancreatic Cancer Treatment Strategies Using a Novel Polytopic Fuzzy Tensor Approach

**DOI:** 10.3390/bioengineering13010002

**Published:** 2025-12-19

**Authors:** Muhammad Bilal, Chaoqian Li, A. K. Alzahrani, A. K. Aljahdali

**Affiliations:** 1 School of Mathematics and Statistics, Yunnan University, Kunming 650106, China; lichaoqian@ynu.cn; 2 Department of Mathematics, King Abdulaziz University, Jeddah 21589, Saudi Arabia; 3 Department of Mathematics, University of Business and Technology, Jeddah 21448, Saudi Arabia

**Keywords:** fuzzy set, Polytopic Fuzzy Set, polytopic fuzzy tensor, cancer treatment, decision making

## Abstract

In response to the growing complexity and uncertainty in real-world decision-making, this study introduces a novel framework based on the polytopic fuzzy tensor (PFT) model, which unifies the geometric structure of polytopes with the representational power of fuzzy tensors. The PFT framework is specifically designed to handle high-dimensional, imprecise, and ambiguous information commonly encountered in multi-criteria group decision-making scenarios. To support this framework, we define a suite of algebraic operations, aggregation mechanisms, and theoretical properties tailored to the PFT environment, with comprehensive mathematical formulations and illustrative validations. The effectiveness of the proposed method is demonstrated through a real-world application involving the evaluation of six pancreatic cancer treatment strategies. These alternatives are assessed against five key criteria: quality of life, side effects, treatment accessibility, cost, and duration. Our results reveal that the PFT-based approach outperforms traditional fuzzy decision-making techniques by delivering more consistent, interpretable, and reliable outcomes under uncertainty. Moreover, comparative analysis confirms the model’s superior ability to handle multidimensional expert evaluations and integrate conflicting information. This research contributes a significant advancement in the field of fuzzy decision science by offering a flexible, theoretically sound, and practically applicable tool for complex decision problems. Future work will focus on improving computational performance, adapting the model for real-time data, and exploring broader interdisciplinary applications.

## 1. Introduction

Pancreatic cancer poses significant challenges in clinical decision-making due to its aggressive progression and limited treatment effectiveness. Medical professionals must consider numerous factors—such as patient quality of life, adverse effects, financial cost, treatment duration, and availability—before recommending a suitable therapeutic strategy. Traditional decision-making methods may fall short when handling such complex, multi-criteria problems. To address this, our study explores the application of advanced multi-criteria decision-making (MCDM) techniques, including GRA, TOPSIS, WASPAS, EDAS, MOORA, and a newly proposed method, for evaluating pancreatic cancer treatment strategies. By incorporating fuzzy-based analysis, the proposed approach aims to offer a more reliable and nuanced evaluation framework to support clinical judgments and optimize patient outcomes.

In modern data analysis, the methodologies used to interpret and process collected information have progressed significantly. However, as datasets become increasingly complex—due to uncertain measurements, incomplete human expertise, or multidimensional relationships—conventional crisp set approaches often fail to capture underlying ambiguity. To address this limitation, advanced mathematical theories have been introduced to enable robust reasoning under uncertainty. Among them, Fuzzy Set (FS) theory, pioneered by Zadeh in 1965 [1], provides a flexible way of expressing partial truths by assigning membership degrees between 0 and 1. This foundational framework has since proven instrumental in numerous domains where imprecision is inherent.

Building upon FS theory, researchers have proposed several enhancements to tackle more sophisticated real-world scenarios. One such development is cut set theory, crucial for analyzing fuzzy matrices (FMs), where Fan and Liu [2,3] contributed significantly by formulating decomposition theorems to improve FM applicability. Anti-fuzzy theory [4,5] later emerged, offering a complementary structure for algebraic modeling in uncertain contexts. Expanding the capabilities of fuzzy modeling to multidimensional spaces, Chen and Lu [6,7] merged fuzzy theory with tensor algebra to propose fuzzy tensors (FTs) and intuitionistic fuzzy tensors (IFTs). These innovations enable the effective representation and analysis of complex, high-order data, making them particularly suitable for modern data science problems.

A further leap in uncertainty modeling was achieved with Atanassov’s introduction of Intuitionistic Fuzzy Sets (IFSs) in 1986 [8], which incorporate both membership and non-membership degrees with the constraint that their sum does not exceed unity. This dual representation allows a more refined modeling of hesitation or indeterminacy in data. Applications of IFS in decision analysis are numerous, particularly through Intuitionistic Fuzzy Matrices (IFMs), which have been widely adopted for practical evaluations. Several researchers have refined decomposition techniques for IFMs: Yuan et al. [9] applied cut sets for structural analysis, Muthuraji and Sriram [10] utilized cut matrices for simplification, Lee and Jeong [11] introduced decompositions into nilpotent and symmetric parts, and Murugadas and Lalitha [12] employed implication operators for algebraic factorization. Recognizing the expressive limitations of IFS, Yager [13] introduced the Pythagorean Fuzzy Set (PFS) in 2013, where the sum of squares of membership and non-membership degrees is constrained to be ≤1. This relaxed condition provides increased flexibility in representing uncertainty, especially in complex multi-criteria decision-making (MCDM) problems.

Meanwhile, tensor-based methodologies have gained prominence in applied mathematics and data analysis, especially since 2005 [14,15]. Tensors generalize matrices to higher dimensions, allowing for richer representations of structured data. Their decomposition—analogous to matrix factorization—has become a vital technique in extracting patterns from multidimensional data. Prominent decomposition methods include Tucker Decomposition [16,17], CP Decomposition [18,19], and Tensor Train Decomposition [20,21]. The origins of tensor decomposition can be traced to Hitchcock’s early work in 1927 [22,23], and continued advancements have enabled wide applications in signal processing, image analysis, and machine learning.

### 1.1. Recent Advances and Relevance to the Present Study

The field of fuzzy decision-making continues to evolve, with recent methodologies providing a compelling foundation for our work. A key advancement is seen in intuitionistic fuzzy Dombi aggregation operators [24], which enhance the representation of hesitation and uncertainty. Their superior ability to fuse complex information has proven valuable in real-world applications, where capturing the nuances of human judgment is critical.

Further expanding the toolkit for decision-makers, quasi-rung orthopair fuzzy sets [25] have been developed for group decision-making. These sets offer a more expressive framework for handling complex, conflicting attributes as demonstrated in practical contexts like selecting optimal sites for electric vehicle charging stations. This highlights their robustness in spatial and multi-factor evaluations.

Addressing the crucial dimension of expert reliability, recent models such as intuitionistic fuzzy confidence-level-based Dombi operators [26] have been introduced. These are particularly useful when the weight or credibility of decision-makers is unknown, enabling effective analysis even with incomplete or highly subjective input.

In a closely related and recent contribution, Bilal et al. [27,28] proposed a hesitant fuzzy tensor-based model and fuzzy soft tensor-based model for group decision-making, specifically applied to wireless network evaluation. This work underscores the growing recognition of tensor structures for managing complex, multidimensional data in a fuzzy context.

Building upon these significant developments, the present study introduces a novel polytopic fuzzy tensor (PFT) framework. Our approach synthesizes concepts from tensor modeling, fuzzy uncertainty, and polytopic geometry. It extends the current state of the art, including the recent hesitant fuzzy tensor model, by offering a richer structural representation for multi-perspective problems, enhanced aggregation flexibility, and a more robust mechanism for modeling indeterminacy across expert evaluations and criteria.

### 1.2. Polytopic Fuzzy Tensor for Pancreatic Cancer Treatment Evaluation

In healthcare, and particularly in oncology, treatment evaluation often involves conflicting criteria and incomplete data. Pancreatic cancer is a prime example where multiple treatment strategies must be assessed under clinical, economic, and patient-specific constraints. However, the uncertainty in patient outcomes, side effects, and therapy effectiveness poses serious challenges to decision-making.

To address this, we propose a polytopic fuzzy tensor (PFT) framework—a novel integration of fuzzy logic, tensor modeling, and polytopic geometry—to facilitate robust and reliable evaluation of pancreatic cancer treatment strategies. Unlike traditional methods, the PFT model allows decision-makers to represent each criterion as a polytopic fuzzy value within a high-dimensional tensor space. This enables the simultaneous capture of imprecision, correlation, and multi-way interactions among decision variables.

The proposed approach utilizes a structured decision-making algorithm that transforms complex and uncertain medical data into a comparative framework for ranking treatment alternatives. By applying the PFT methodology to real-world data, this study demonstrates how robust, uncertainty-aware evaluations can be achieved, leading to more informed and confident clinical recommendations.

### 1.3. Research Gaps

Despite significant advancements in fuzzy multi-criteria decision-making (MCDM) methods, several critical research gaps remain that motivate the development of the proposed polytopic fuzzy tensor (PFT) framework:

1. Lack of Integration between Fuzzy Logic and High-Dimensional Structures: Existing fuzzy decision-making models are predominantly matrix-based and lack the capacity to represent higher-order interdependencies among criteria and alternatives in a structured, multidimensional form.
2. Inability to Incorporate Geometric Constraints in Fuzzy Models: Conventional fuzzy and tensor-based models do not embed geometric feasibility conditions such as polytopic boundaries, which are vital for ensuring valid and interpretable decision spaces in constrained environments.
3. Insufficient Handling of Multi-Expert, Multi-Criteria Evaluations Under Uncertainty: There is a lack of robust frameworks that can simultaneously process evaluations from multiple experts across multiple criteria while capturing the uncertainty and imprecision inherent in such complex settings.
4. Limited Application of Advanced Fuzzy Models in Pancreatic Cancer Treatment Evaluation: Although fuzzy MCDM techniques have been applied in healthcare, few studies focus specifically on the evaluation of pancreatic cancer treatments, which involve complex trade-offs, uncertain information, and expert disagreement.
5. Reduced Robustness and Interpretability in Traditional MCDM Techniques: Many classical decision-making approaches struggle with producing robust and interpretable outcomes when faced with highly uncertain and structurally complex data, particularly in multidimensional decision scenarios.

### 1.4. Motivation and Goals

In today’s complex decision-making environments, especially in critical sectors such as healthcare, transportation, and environmental management, decision-makers frequently encounter uncertain, imprecise, and interrelated information. Traditional fuzzy models, while effective in handling vagueness, often fall short in capturing the multidimensional structure and geometric constraints present in real-world problems. Similarly, existing tensor-based approaches may lack the flexibility to express fuzziness in a form that is interpretable and operational for decision-makers.

The motivation behind this research arises from the need to integrate multidimensional data representation, fuzzy logic, and geometric feasibility within a unified decision-making model. Real-life problems such as evaluating cancer treatment strategies involve multiple experts, criteria, and interdependencies—making them ideal candidates for a model that can handle high-order fuzzy data and preserve logical and spatial structure.

To address these challenges, this study introduces the polytopic fuzzy tensor (PFT) framework—a novel mathematical and decision-making model that encapsulates the following core goals:

- To develop a new tensor-based structure capable of modeling multidimensional fuzzy information under polytopic constraints;
- To define and validate essential algebraic operations that preserve both fuzzy semantics and polytopic feasibility;
- To construct a comprehensive decision-making algorithm using PFT that identifies ideal and anti-ideal solutions and ranks alternatives accordingly;
- To demonstrate the real-world applicability of the PFT model through a healthcare case study focused on evaluating pancreatic cancer treatment options under uncertainty.

As demonstrated in our comparative analysis, the PFT framework outperforms traditional approaches such as TOPSIS, GRA, and EDAS in delivering stable and reliable treatment rankings in the context of pancreatic cancer decision-making. This confirms the effectiveness and novelty of our method in modeling high-dimensional uncertainty and supporting more informed decisions.

### 1.5. Contribution

This article makes several significant contributions to the field of fuzzy decision-making and its applications in healthcare evaluation. The key advancements are summarized as follows:

- **Novel Framework Development:** We introduce a *polytopic fuzzy tensor* (PFT) structure that synergistically combines the geometric interpretability of polytopes with the multidimensional representation capability of fuzzy tensors. This integration provides a robust mathematical foundation for handling complex, uncertain data in decision-making processes.
- **Theoretical Advancements:** The paper establishes essential algebraic operations, properties, and aggregation operators specifically designed for the PFT framework. Rigorous mathematical proofs and illustrative examples validate the theoretical soundness and practical applicability of the proposed approach.
- **Healthcare Decision-Making Application:** We demonstrate the real-world utility of the PFT framework through an in-depth case study evaluating six pancreatic cancer treatment strategies. The model effectively incorporates multiple clinically relevant criteria—including quality of life, side effects, cost, and treatment duration—providing a comprehensive assessment tool for medical decision-makers.
- **Comparative Validation:** Through systematic comparison with existing decision-making methods, we empirically demonstrate the superior robustness and accuracy of our approach in handling multidimensional uncertainty. The results show significant improvements in both decision quality and interpretability.
- **Practical Implications:** The proposed framework offers medical practitioners and healthcare policymakers a flexible, intuitive tool for treatment evaluation under uncertainty. The method’s interpretability features facilitate transparent communication of complex decision rationales to stakeholders.

These contributions collectively advance the state of the art in uncertainty-aware decision-making, with particular significance for complex medical evaluations where multidimensional criteria and expert judgments must be balanced under imperfect information.

### 1.6. Organization

The organization of this paper is as follows: Section 1 presents the introduction. Section 2 is about the basic definitions. Section 3 presents a new approach to polytopic fuzzy tensor with examples, basic operations, basic properties, basic theorems, De Morgans laws, and aggregation operators. Section 4 presents a proposed decision-making algorithm, problem statement, and solution of application by using the proposed approach. Section 5 presents the results and discussions. Section 6 presents a computational complexity and scalability analysis. Section 7 presents a comparison analysis. Section 8 presents a sensitivity analysis. Section 9 presents the advantages and limitations. And at the end in Section 10, we discuss the conclusion, implications, and future research directions.

## 2. Preliminaries

The rapid growth of multi-criteria decision-making (MCDM) methodologies over the past decade has generated a diverse landscape of models designed to handle uncertainty, expert subjectivity, and complex evaluation structures. While classical fuzzy MCDM techniques such as Fuzzy AHP, Fuzzy TOPSIS, and Fuzzy ELECTRE have demonstrated strong applicability across various domains, their reliance on matrix-based formulations and repeated normalization or comparison cycles often limits scalability and flexibility. Recent studies have explored enriched uncertainty representations—such as hesitant fuzzy sets, interval-valued fuzzy systems, and tensor-based frameworks—to overcome these challenges. In this section, we review the most relevant developments in the literature and provide the foundational concepts necessary for understanding the proposed PFT model. This overview establishes the methodological context of our work and highlights the motivations for adopting a higher-order fuzzy representation in decision-making.

**Definition** **1**  ([1])**.** *A **Fuzzy Set (FS)** A in a universe X is defined as*

$$A=\{\left(x,\mu_{A}\left(x\right)\right)|x\in X\}$$

*where $\mu_{A}:X\to \left[0,1\right]$ is the membership function indicating the degree of membership of each element x∈X in the set A.*

**Definition** **2**  ([14,15])**.** *A **Tensor** is a multidimensional array of order n represented by*

$$𝒯\in R^{I_{1}\times I_{2}\times \cdots \times I_{n}},$$

*where $I_{k}$ denotes the size along the k-th dimension (mode). Tensors generalize matrices to higher dimensions and are used in many applications, including data analysis and signal processing.*

**Definition** **3**  ([6])**.** *A **Fuzzy Tensor (FT)** is a higher-order array in which each element is a fuzzy number or value in* [0, 1]*. It extends the concept of fuzzy matrices to multidimensional domains and is useful in representing complex uncertain systems.*

**Definition** **4**  ([29])**.** *A **Polytopic Fuzzy Number (PFN)** is a fuzzy number whose membership function is defined over a polytope in $R^{n}$. It is described as*

$$PFN=\{\left(x,\mu \left(x\right)\right)|x\in P\subset R^{n},\mu \left(x\right)\in \left[0,1\right]\}$$

*where P is a convex polytope, and μ(x) represents the degree of membership of the point x in the polytope.*

**Definition** **5**  ([29])**.** *A **Polytopic Fuzzy Set (PFS)** is a generalization of a fuzzy set where the universe of discourse is a convex polytope in a multidimensional space, and each point in the polytope is associated with a fuzzy membership value:*

$$A=\{\left(x,\mu_{A}\left(x\right)\right)|x\in P\subset R^{n},\mu_{A}\left(x\right)\in \left[0,1\right]\}.$$

## 3. Polytopic Fuzzy Tensor

This section introduces the core concept of the *polytopic fuzzy tensor* (PFT), the novel mathematical structure at the heart of our proposed framework. We begin by formally defining a PFT as a higher-order construct that encapsulates multiple fuzzy tensors, each representing data or evaluations from a different perspective, such as various experts or systems. This design allows the model to naturally capture and handle the inherent uncertainty, imprecision, and variability found in complex, multidimensional decision-making scenarios. Following the definition, we provide several illustrative examples to ground the concept, demonstrating its application across different domains from expert evaluation to temporal analysis. Cite Appendix A at operations, properties and Theorems section. As examples are from these sections in Appendix A.

**Definition** **6.**  *Let $X=\{x_{1},x_{2},\ldots ,x_{n}\}$ be a finite universe and let I={1,2,…,m} be an index set representing multiple perspectives, decision makers, or systems. A **polytopic fuzzy tensor*** *𝒫 of order k and dimension n is defined as a collection of fuzzy tensors from multiple viewpoints such that*

$$𝒫=\left\{A^{\left(i\right)}\in {\left[0,1\right]}^{n\times n\times \cdots \times n}|i\in I\right\},\;\mathrm{with}\;k\;\mathrm{modes}$$

*Each $A^{\left(i\right)}$ is a fuzzy tensor of order k with entries $\mu_{j_{1}j_{2}\ldots j_{k}}^{\left(i\right)}\in \left[0,1\right]$, where the superscript (i) indicates the i-th perspective or decision maker.*

*A polytopic fuzzy tensor can thus be viewed as a higher-order structure integrating multiple fuzzy tensors to capture uncertainty, imprecision, and variability across multiple dimensions and viewpoints.*

**Example** **1.**  ***Polytopic Fuzzy Tensor of Order 2 (Matrix Form)***

*Consider a system evaluated by three experts (i=1,2,3) over two attributes. Each expert provides a fuzzy relation matrix:*

$$𝒫=\left\{A^{\left(1\right)}=\left[\begin{array}{cc} 0.9 & 0.6\\ 0.4 & 0.8 \end{array}\right],\;A^{\left(2\right)}=\left[\begin{array}{cc} 0.7 & 0.5\\ 0.6 & 0.9 \end{array}\right],\;A^{\left(3\right)}=\left[\begin{array}{cc} 0.8 & 0.7\\ 0.5 & 0.6 \end{array}\right]\right\}$$

*Here, 𝒫 is a polytopic fuzzy tensor of order 2 and dimension 2, aggregating fuzzy matrices from three perspectives.*

**Example** **2.**  ***Multi-Expert Multi-Symptom Diagnosis Assessment***

*Consider a medical diagnostic scenario involving three symptoms ($S_{1},S_{2},S_{3}$), two possible diseases ($D_{1},D_{2}$), and two medical experts providing fuzzy assessments across three severity levels ($L_{1},L_{2},L_{3}$). Each expert’s evaluation is represented as a third-order fuzzy tensor $A^{\left(i\right)}$ of shape 3×2×3, where*
$$A_{s,d,l}^{\left(i\right)}=\mathrm{Expert}\;i^{\prime }s\;\mathrm{belief}\;\mathrm{that}\;\mathrm{symptom}\;s\;\mathrm{indicates}\;\mathrm{disease}\;d\;\mathrm{at}\;\mathrm{severity}\;\mathrm{level}\;l.$$

*For instance, let the two experts provide the following fuzzy tensors:*

$$A^{\left(1\right)}\left(\cdot ,\cdot ,1\right)=\left[\begin{array}{cc} 0.8 & 0.2\\ 0.6 & 0.4\\ 0.5 & 0.3 \end{array}\right],\;A^{\left(1\right)}\left(\cdot ,\cdot ,2\right)=\left[\begin{array}{cc} 0.7 & 0.5\\ 0.5 & 0.6\\ 0.4 & 0.4 \end{array}\right],\;A^{\left(1\right)}\left(\cdot ,\cdot ,3\right)=\left[\begin{array}{cc} 0.6 & 0.7\\ 0.4 & 0.5\\ 0.3 & 0.6 \end{array}\right]$$

$$A^{\left(2\right)}\left(\cdot ,\cdot ,1\right)=\left[\begin{array}{cc} 0.9 & 0.3\\ 0.7 & 0.5\\ 0.6 & 0.4 \end{array}\right],\;A^{\left(2\right)}\left(\cdot ,\cdot ,2\right)=\left[\begin{array}{cc} 0.8 & 0.6\\ 0.6 & 0.7\\ 0.5 & 0.5 \end{array}\right],\;A^{\left(2\right)}\left(\cdot ,\cdot ,3\right)=\left[\begin{array}{cc} 0.7 & 0.8\\ 0.5 & 0.6\\ 0.4 & 0.7 \end{array}\right]$$

*Thus, the polytopic fuzzy tensor $𝒫=\{A^{\left(1\right)},A^{\left(2\right)}\}$ captures diagnostic uncertainty and inter-expert variability in a clinically meaningful multidimensional format.*

**Example** **3.**  ***Application in Multi-Expert Medical Diagnosis***

*Assume a medical diagnosis scenario with 3 symptoms, 2 diseases, and 2 experts providing fuzzy evaluations. Each tensor $A^{\left(i\right)}$ is of order 3:*

$$A_{ijk}^{\left(1\right)}=\mathrm{Expert}\;1^{\prime }s\;\mathrm{belief}\;\mathrm{that}\;\mathrm{symptom}\;i\;\mathrm{indicates}\;\mathrm{disease}\;j\;\mathrm{at}\;\mathrm{severity}\;k$$

$$A_{ijk}^{\left(2\right)}=\mathrm{Expert}\;2^{\prime }s\;\mathrm{belief}$$

*Suppose*

$$A^{\left(1\right)}\left(:,:,1\right)=\left[\begin{array}{cc} 0.9 & 0.3\\ 0.7 & 0.5\\ 0.6 & 0.4 \end{array}\right],\;A^{\left(1\right)}\left(:,:,2\right)=\left[\begin{array}{cc} 0.8 & 0.6\\ 0.5 & 0.7\\ 0.4 & 0.3 \end{array}\right]$$

$$A^{\left(2\right)}\left(:,:,1\right)=\left[\begin{array}{cc} 0.7 & 0.2\\ 0.6 & 0.6\\ 0.5 & 0.3 \end{array}\right],\;A^{\left(2\right)}\left(:,:,2\right)=\left[\begin{array}{cc} 0.9 & 0.7\\ 0.4 & 0.8\\ 0.3 & 0.2 \end{array}\right]$$

*This $𝒫=\{A^{\left(1\right)},A^{\left(2\right)}\}$ is a polytopic fuzzy tensor capturing expert disagreement.*

**Example** **4.**  ***Multi-Sensor Multi-Time Patient Recovery Monitoring***

*Consider a post-operative monitoring system that tracks two vital signs (e.g., heart rate variability and oxygen saturation) across three consecutive time intervals ($t_{1},t_{2},t_{3}$), using two independent medical sensors. Each sensor provides a fuzzy assessment of physiological stability, where values closer to 1 indicate higher stability.*

*Let $A^{\left(1\right)}$ and $A^{\left(2\right)}$ represent the fuzzy tensors from Sensor 1 and Sensor 2, respectively, each of order 3 with dimensions 2×2×3 (vital signs × measurement sites × time intervals).*

*For instance,*
$$A^{\left(1\right)}\left(\cdot ,\cdot ,1\right)=\left[\begin{array}{cc} 0.8 & 0.6\\ 0.7 & 0.5 \end{array}\right],\;A^{\left(1\right)}\left(\cdot ,\cdot ,2\right)=\left[\begin{array}{cc} 0.7 & 0.5\\ 0.6 & 0.4 \end{array}\right],\;A^{\left(1\right)}\left(\cdot ,\cdot ,3\right)=\left[\begin{array}{cc} 0.6 & 0.4\\ 0.5 & 0.3 \end{array}\right]$$
$$A^{\left(2\right)}\left(\cdot ,\cdot ,1\right)=\left[\begin{array}{cc} 0.7 & 0.5\\ 0.6 & 0.4 \end{array}\right],\;A^{\left(2\right)}\left(\cdot ,\cdot ,2\right)=\left[\begin{array}{cc} 0.6 & 0.4\\ 0.5 & 0.3 \end{array}\right],\;A^{\left(2\right)}\left(\cdot ,\cdot ,3\right)=\left[\begin{array}{cc} 0.5 & 0.3\\ 0.4 & 0.2 \end{array}\right]$$

*Here, $𝒫=\{A^{\left(1\right)},A^{\left(2\right)}\}$ is a polytopic fuzzy tensor capturing temporal evolution and inter-sensor variability in a clinically meaningful monitoring context. This structure allows for integrated analysis of patient recovery trends under uncertainty.*

### 3.1. Operations on Polytopic Fuzzy Tensors

To develop a robust algebraic framework for polytopic fuzzy tensors (PFTs), it is essential to define fundamental operations that extend fuzzy logic and tensor algebra into the polytopic context. The following operations are defined element-wise across corresponding tensors.

Let $𝒫=\{A^{\left(1\right)},A^{\left(2\right)},\ldots ,A^{\left(m\right)}\}$ and $Q=\{B^{\left(1\right)},B^{\left(2\right)},\ldots ,B^{\left(m\right)}\}$ be two polytopic fuzzy tensors of the same order and dimensions.

**Definition** **7**  **(Addition).** *Addition of PFTs can be defined as*

$${\left(A^{\left(i\right)}⊕B^{\left(i\right)}\right)}_{j_{1}j_{2}\ldots j_{k}}=\min\left(1,A_{j_{1}j_{2}\ldots j_{k}}^{\left(i\right)}+B_{j_{1}j_{2}\ldots j_{k}}^{\left(i\right)}\right)$$

**Definition** **8**  **(Multiplication (Bounded Product)).** *The multiplication of PFTs is defined as*

$${\left(A^{\left(i\right)}⊗B^{\left(i\right)}\right)}_{j_{1}j_{2}\ldots j_{k}}=\min\left(A_{j_{1}j_{2}\ldots j_{k}}^{\left(i\right)},B_{j_{1}j_{2}\ldots j_{k}}^{\left(i\right)}\right)$$

**Definition** **9**  **(Hadamard Product (Element-wise Multiplication)).** *It is defined as the product of corresponding elements*

$${\left(A^{\left(i\right)}\circ B^{\left(i\right)}\right)}_{j_{1}j_{2}\ldots j_{k}}=A_{j_{1}j_{2}\ldots j_{k}}^{\left(i\right)}\cdot B_{j_{1}j_{2}\ldots j_{k}}^{\left(i\right)}$$

**Definition** **10**  **(Complement).** *The complement of PFT is computed as*

$${\left(\neg A^{\left(i\right)}\right)}_{j_{1}j_{2}\ldots j_{k}}=1-A_{j_{1}j_{2}\ldots j_{k}}^{\left(i\right)}$$

**Definition** **11**  **(Scalar Multiplication).** *Let λ∈[0,1], then scalar multiplication is*

$${\left(\lambda \cdot A^{\left(i\right)}\right)}_{j_{1}j_{2}\ldots j_{k}}=\lambda \cdot A_{j_{1}j_{2}\ldots j_{k}}^{\left(i\right)}$$

**Definition** **12**  **(α-Cut Tensor).** *For a threshold α∈[0,1], the α-cut of a fuzzy tensor is a crisp tensor:*

$${\left(A^{\left(i\right)}\right)}_{\alpha }=\left\{\begin{array}{cc} 1 & \mathrm{if}\;A_{j_{1}j_{2}\ldots j_{k}}^{\left(i\right)}\geq \alpha \\ 0 & \mathrm{otherwise} \end{array}\right.$$

**Definition** **13**  **(Union and Intersection of Polytopic Fuzzy Tensors).** *For two PFTs of the same shape,*

$${\left(A^{\left(i\right)}\cup B^{\left(i\right)}\right)}_{j_{1}j_{2}\ldots j_{k}}=\max\left(A_{j_{1}j_{2}\ldots j_{k}}^{\left(i\right)},B_{j_{1}j_{2}\ldots j_{k}}^{\left(i\right)}\right)$$

$${\left(A^{\left(i\right)}\cap B^{\left(i\right)}\right)}_{j_{1}j_{2}\ldots j_{k}}=\min\left(A_{j_{1}j_{2}\ldots j_{k}}^{\left(i\right)},B_{j_{1}j_{2}\ldots j_{k}}^{\left(i\right)}\right)$$

#### 3.1.1. Interpretation of Operations in the PFT Framework

In the proposed polytopic fuzzy tensor (PFT) framework, conventional fuzzy operations such as bounded sum, minimum, maximum, and scalar multiplication are reinterpreted to accommodate the intrinsic characteristics of polytopic geometry and multidimensional fuzzy logic. Unlike traditional approaches where these operations are applied element-wise with no spatial constraints, in PFT these operations are constrained within the multidimensional geometric structure of a polytope. For instance, the bounded sum is modified to ensure that the aggregated fuzzy values do not violate the polytopic boundary conditions or exceed the unit interval, maintaining logical consistency and structural feasibility. Similarly, the min and max operations are adapted to preserve the component-wise order while respecting inter-dimensional dependencies represented by the tensor structure. Scalar multiplication within the PFT framework adjusts the magnitude of the tensor elements while retaining the directional properties defined by the polytope. These tailored interpretations are critical for ensuring that operations yield results that are both fuzzily meaningful and geometrically valid, thereby enhancing the robustness and applicability of the PFT model in uncertain multi-criteria decision environments.

#### 3.1.2. Interpretive Notes on PFT Operations

The above operations form the algebraic backbone of the polytopic fuzzy tensor framework, allowing for comprehensive modeling, combination, and transformation of fuzzy multi-way data. These operations support the further development of decision-making algorithms, inference mechanisms, and optimization techniques in high-dimensional, uncertain environments.

### 3.2. Algebraic Properties of Polytopic Fuzzy Tensors

In this section, we explore fundamental algebraic properties satisfied by polytopic fuzzy tensors (PFTs). Let $𝒫=\{A^{\left(1\right)},A^{\left(2\right)},\ldots ,A^{\left(m\right)}\}$ denote a polytopic fuzzy tensor, where each $A^{\left(i\right)}$ is a fuzzy tensor of order *k* with membership values in [0,1].

**Property** **1**  **(Boundedness).** *If $𝒫=\{A^{\left(1\right)},A^{\left(2\right)},\ldots ,A^{\left(m\right)}\}$ is a polytopic fuzzy tensor where each $A^{\left(i\right)}$ is a fuzzy tensor of order k with dimension n, then for all i∈{1,…,m} and all index tuples $\left(j_{1},j_{2},\ldots ,j_{k}\right)$ with $1\leq j_{r}\leq n$, the following inequality holds:*

$$0\leq A_{j_{1}j_{2}\ldots j_{k}}^{\left(i\right)}\leq 1.$$

**Proof.**  Let $𝒫=\{A^{\left(1\right)},A^{\left(2\right)},\ldots ,A^{\left(m\right)}\}$ be a polytopic fuzzy tensor as defined in Definition 6, where each $A^{\left(i\right)}$ is a fuzzy tensor of order *k*.

1. **By Definition 3 (Fuzzy Tensor):** A fuzzy tensor is a higher-order array in which every element is a fuzzy number or a value in [0,1]. Therefore, for each $A^{\left(i\right)}$ and any index tuple $\left(j_{1},j_{2},\ldots ,j_{k}\right)$:
  $$A_{j_{1}j_{2}\ldots j_{k}}^{\left(i\right)}\in \left[0,1\right].$$
2. **By Definition 6 (Polytopic Fuzzy Tensor):** A polytopic fuzzy tensor 𝒫 is a finite collection of such fuzzy tensors. Therefore, for every i∈{1,…,m}, $A^{\left(i\right)}$ satisfies
  $$0\leq A_{j_{1}j_{2}\ldots j_{k}}^{\left(i\right)}\leq 1.$$
3. **Thus, for all i and all index tuples:** Since each $A^{\left(i\right)}$ in 𝒫 is a fuzzy tensor and inherits the boundedness from Definition 3, it follows directly that
  $$0\leq A_{j_{1}j_{2}\ldots j_{k}}^{\left(i\right)}\leq 1\;\forall i,\forall j_{1},\ldots ,j_{k}.$$

   □

**Property** **2**  **(Monotonicity).** *If $A_{j_{1}\ldots j_{k}}^{\left(i\right)}\leq B_{j_{1}\ldots j_{k}}^{\left(i\right)}$ for all i=1,…,m and for all indices $j_{1},\ldots ,j_{k}$, then for any monotonic aggregation operator $F:{\left[0,1\right]}^{m}\to \left[0,1\right]$*

$$F\left(A_{j_{1}\ldots j_{k}}^{\left(1\right)},\ldots ,A_{j_{1}\ldots j_{k}}^{\left(m\right)}\right)\leq F\left(B_{j_{1}\ldots j_{k}}^{\left(1\right)},\ldots ,B_{j_{1}\ldots j_{k}}^{\left(m\right)}\right).$$

**Proof.**  Let ${𝒫}_{A}=\left\{A^{\left(1\right)},\ldots ,A^{\left(m\right)}\right\}$ and ${𝒫}_{B}=\left\{B^{\left(1\right)},\ldots ,B^{\left(m\right)}\right\}$ be two polytopic fuzzy tensors such that

$$A_{j_{1}\ldots j_{k}}^{\left(i\right)}\leq B_{j_{1}\ldots j_{k}}^{\left(i\right)}\;\mathrm{for}\;\mathrm{all}\;i\;\mathrm{and}\;\mathrm{all}\;j_{1},\ldots ,j_{k}.$$

Let $F:{\left[0,1\right]}^{m}\to \left[0,1\right]$ be a monotonic aggregation operator. By definition of monotonicity, if $a_{i}\leq b_{i}$ for all i=1,…,m, then

$$F\left(a_{1},\ldots ,a_{m}\right)\leq F\left(b_{1},\ldots ,b_{m}\right).$$

Now, for any fixed index tuple $\left(j_{1},\ldots ,j_{k}\right)$, consider the vectors

$$a=\left(A_{j_{1}\ldots j_{k}}^{\left(1\right)},\ldots ,A_{j_{1}\ldots j_{k}}^{\left(m\right)}\right),\;b=\left(B_{j_{1}\ldots j_{k}}^{\left(1\right)},\ldots ,B_{j_{1}\ldots j_{k}}^{\left(m\right)}\right).$$

Since $A_{j_{1}\ldots j_{k}}^{\left(i\right)}\leq B_{j_{1}\ldots j_{k}}^{\left(i\right)}$ for all *i*, we have $a_{i}\leq b_{i}$ for all *i*. By the monotonicity of F,

F(a)≤F(b).

This holds for every $\left(j_{1},\ldots ,j_{k}\right)$, and therefore

$$F{\left(A\right)}_{j_{1}\ldots j_{k}}\leq F{\left(B\right)}_{j_{1}\ldots j_{k}}\;\mathrm{for}\;\mathrm{all}\;\mathrm{indices}\;j_{1},\ldots ,j_{k}.$$

Hence, F(A)≤F(B) holds element-wise across the entire aggregated tensor, which completes the proof.    □

**Property** **3**  **(Idempotency).** *Let $𝒫=\{A^{\left(1\right)},A^{\left(2\right)},\ldots ,A^{\left(m\right)}\}$ be a polytopic fuzzy tensor. Then the element-wise union and intersection of 𝒫 with itself satisfy*

𝒫∪𝒫=𝒫,𝒫∩𝒫=𝒫.

**Proof.**  Let $𝒫=\{A^{\left(1\right)},\ldots ,A^{\left(m\right)}\}$ be a polytopic fuzzy tensor as defined in Definition 6, where each $A^{\left(i\right)}$ is a fuzzy tensor of order *k* with components in [0,1].

1. **Union operation ∪ (Definition 13, extended to PFTs):** The union of two identical polytopic fuzzy tensors is performed component-wise across corresponding tensors $A^{\left(i\right)}$:
  $${\left(𝒫\cup 𝒫\right)}^{\left(i\right)}=A^{\left(i\right)}\cup A^{\left(i\right)}.$$
  For any component at position $\left(j_{1},\ldots ,j_{k}\right)$ and for each i=1,…,m:
  $${\left(A^{\left(i\right)}\cup A^{\left(i\right)}\right)}_{j_{1}\ldots j_{k}}=\max\left(A_{j_{1}\ldots j_{k}}^{\left(i\right)},A_{j_{1}\ldots j_{k}}^{\left(i\right)}\right)=A_{j_{1}\ldots j_{k}}^{\left(i\right)}.$$
  Since this holds for all *i* and all components, we have
  $$𝒫\cup 𝒫=\{A^{\left(1\right)},\ldots ,A^{\left(m\right)}\}=𝒫.$$
2. **Intersection operation ∩ (Definition 13, extended to PFTs):** Similarly, the intersection is defined component-wise:
  $${\left(𝒫\cap 𝒫\right)}^{\left(i\right)}=A^{\left(i\right)}\cap A^{\left(i\right)}.$$
  For any component at position $\left(j_{1},\ldots ,j_{k}\right)$ and for each i=1,…,m,
  $${\left(A^{\left(i\right)}\cap A^{\left(i\right)}\right)}_{j_{1}\ldots j_{k}}=\min\left(A_{j_{1}\ldots j_{k}}^{\left(i\right)},A_{j_{1}\ldots j_{k}}^{\left(i\right)}\right)=A_{j_{1}\ldots j_{k}}^{\left(i\right)}.$$
  Therefore,
  $$𝒫\cap 𝒫=\{A^{\left(1\right)},\ldots ,A^{\left(m\right)}\}=𝒫.$$

Hence, the idempotency property holds for polytopic fuzzy tensors under both union and intersection operations.    □

**Property** **4**  **(Reflexivity).** *Let $𝒫=\{A^{\left(1\right)},A^{\left(2\right)},\ldots ,A^{\left(m\right)}\}$ be a polytopic fuzzy tensor. Then 𝒫 is comparable to itself under the element-wise partial order, and the union with itself yields the original tensor:*

𝒫≤𝒫and𝒫∪𝒫=𝒫.

**Proof.**  Let $𝒫=\{A^{\left(1\right)},\ldots ,A^{\left(m\right)}\}$ be a polytopic fuzzy tensor as defined in Definition 6, where each $A^{\left(i\right)}$ is a fuzzy tensor of order *k* with components in [0,1].

1. **Self-comparability (𝒫≤𝒫):** The partial order ≤ for polytopic fuzzy tensors is defined component-wise across corresponding tensors. That is, for each i=1,…,m and all index tuples $\left(j_{1},\ldots ,j_{k}\right)$:
  $$𝒫\leq 𝒫\;\mathrm{iff}\;A_{j_{1}\ldots j_{k}}^{\left(i\right)}\leq A_{j_{1}\ldots j_{k}}^{\left(i\right)}.$$
  For any real number a∈[0,1], we have a≤a. Since each component $A_{j_{1}\ldots j_{k}}^{\left(i\right)}\in \left[0,1\right]$, it follows that
  $$A_{j_{1}\ldots j_{k}}^{\left(i\right)}\leq A_{j_{1}\ldots j_{k}}^{\left(i\right)}\;\mathrm{for}\;\mathrm{all}\;i,j_{1},\ldots ,j_{k}.$$
  Therefore, by the component-wise definition of the order, 𝒫≤𝒫.
2. **Union with itself (𝒫∪𝒫=𝒫):** The union operation ∪ for polytopic fuzzy tensors is defined element-wise (Definition 13). For each i=1,…,m and each component $\left(j_{1},\ldots ,j_{k}\right)$,
  $${\left(𝒫\cup 𝒫\right)}_{j_{1}\ldots j_{k}}^{\left(i\right)}=\max\left(A_{j_{1}\ldots j_{k}}^{\left(i\right)},A_{j_{1}\ldots j_{k}}^{\left(i\right)}\right).$$
  Since max(a,a)=a for any real number *a*, we have
  $$\max\left(A_{j_{1}\ldots j_{k}}^{\left(i\right)},A_{j_{1}\ldots j_{k}}^{\left(i\right)}\right)=A_{j_{1}\ldots j_{k}}^{\left(i\right)}.$$
  Hence, for all *i* and all components:
  $${\left(𝒫\cup 𝒫\right)}^{\left(i\right)}=A^{\left(i\right)}.$$
  Therefore, 𝒫∪𝒫=𝒫.

Thus, both reflexivity conditions hold for any polytopic fuzzy tensor 𝒫.    □

**Property** **5**  **(Convexity).** *Let ${𝒫}_{A}=\left\{A^{\left(1\right)},\ldots ,A^{\left(m\right)}\right\}$ and ${𝒫}_{B}=\left\{B^{\left(1\right)},\ldots ,B^{\left(m\right)}\right\}$ be two polytopic fuzzy tensors of the same order and dimensions. For any λ∈[0,1], the convex combination defined as*

$${𝒫}_{C}=\left\{\lambda \cdot A^{\left(i\right)}+\left(1-\lambda \right)\cdot B^{\left(i\right)}|i=1,\ldots ,m\right\}$$

*is also a polytopic fuzzy tensor. Specifically, every component satisfies*

$$0\leq C_{j_{1}\ldots j_{k}}^{\left(i\right)}\leq 1\;\mathrm{for}\;\mathrm{all}\;i,j_{1},\ldots ,j_{k}.$$

**Proof.**  Let ${𝒫}_{A}$ and ${𝒫}_{B}$ be polytopic fuzzy tensors as given, and let λ∈[0,1]. Define

$$C^{\left(i\right)}=\lambda \cdot A^{\left(i\right)}+\left(1-\lambda \right)\cdot B^{\left(i\right)}\;\mathrm{for}\;\mathrm{each}\;i=1,\ldots ,m.$$

1. **Boundedness of components:** Since $A^{\left(i\right)}$ and $B^{\left(i\right)}$ are fuzzy tensors, by Definition 3 each of their components lies in [0,1]:
  $$0\leq A_{j_{1}\ldots j_{k}}^{\left(i\right)}\leq 1,\;0\leq B_{j_{1}\ldots j_{k}}^{\left(i\right)}\leq 1.$$
2. **Convex combination preserves bounds:** For any fixed indices $i,j_{1},\ldots ,j_{k}$, let $a=A_{j_{1}\ldots j_{k}}^{\left(i\right)}$ and $b=B_{j_{1}\ldots j_{k}}^{\left(i\right)}$. Then,
  c=λa+(1−λ)b.
  Because 0≤a,b≤1 and 0≤λ≤1, the value *c* is a convex combination of two numbers in [0,1]. Hence:
  0≤c≤1.
  This holds for all components, so
  $$0\leq C_{j_{1}\ldots j_{k}}^{\left(i\right)}\leq 1\;\forall i,j_{1},\ldots ,j_{k}.$$
3. **Preservation of tensor structure:** Each $C^{\left(i\right)}$ is a linear combination of fuzzy tensors $A^{\left(i\right)}$ and $B^{\left(i\right)}$, and therefore retains the same order and dimensions. Since all entries remain in [0,1], each $C^{\left(i\right)}$ is a fuzzy tensor.
4. **Polytopic fuzzy tensor formation:** The set ${𝒫}_{C}=\left\{C^{\left(1\right)},\ldots ,C^{\left(m\right)}\right\}$ is a finite collection of fuzzy tensors, each satisfying the boundedness condition. By Definition 6, ${𝒫}_{C}$ is therefore a polytopic fuzzy tensor.

Thus, the convex combination of two polytopic fuzzy tensors yields another polytopic fuzzy tensor with all components bounded within [0,1].    □

**Property** **6**  **(Associativity of Union and Intersection).** *Let ${𝒫}_{A}=\left\{A^{\left(1\right)},\ldots ,A^{\left(m\right)}\right\}$, ${𝒫}_{B}=\left\{B^{\left(1\right)},\ldots ,B^{\left(m\right)}\right\}$, and ${𝒫}_{C}=\left\{C^{\left(1\right)},\ldots ,C^{\left(m\right)}\right\}$ be three polytopic fuzzy tensors of the same order and dimensions. Then the union and intersection operations are associative:*

$$\left({𝒫}_{A}\cup {𝒫}_{B}\right)\cup {𝒫}_{C}={𝒫}_{A}\cup \left({𝒫}_{B}\cup {𝒫}_{C}\right),$$

*and*

$$\left({𝒫}_{A}\cap {𝒫}_{B}\right)\cap {𝒫}_{C}={𝒫}_{A}\cap \left({𝒫}_{B}\cap {𝒫}_{C}\right).$$

**Proof.**  The union and intersection of polytopic fuzzy tensors are defined component-wise according to Definition 13. We prove associativity for the union operation; the proof for intersection follows analogously.

1. **Union associativity:** Let ${𝒫}_{U}=\left({𝒫}_{A}\cup {𝒫}_{B}\right)\cup {𝒫}_{C}$ and ${𝒫}_{V}={𝒫}_{A}\cup \left({𝒫}_{B}\cup {𝒫}_{C}\right)$. For any i=1,…,m and any index tuple $\left(j_{1},\ldots ,j_{k}\right)$, we compute the components:
  $$\begin{array}{cc} {{𝒫}_{U}^{\left(i\right)}}_{j_{1}\ldots j_{k}} & =\max\left(\max\left(A_{j_{1}\ldots j_{k}}^{\left(i\right)},B_{j_{1}\ldots j_{k}}^{\left(i\right)}\right),C_{j_{1}\ldots j_{k}}^{\left(i\right)}\right),\\ {{𝒫}_{V}^{\left(i\right)}}_{j_{1}\ldots j_{k}} & =\max\left(A_{j_{1}\ldots j_{k}}^{\left(i\right)},\max\left(B_{j_{1}\ldots j_{k}}^{\left(i\right)},C_{j_{1}\ldots j_{k}}^{\left(i\right)}\right)\right). \end{array}$$
  The ordinary max function on real numbers is associative: for any a,b,c∈R,
  max(max(a,b),c)=max(a,max(b,c)).
  Since each component $A_{j_{1}\ldots j_{k}}^{\left(i\right)},B_{j_{1}\ldots j_{k}}^{\left(i\right)},C_{j_{1}\ldots j_{k}}^{\left(i\right)}$ is a real number in [0,1], it follows that
  𝒫U(i)=j1…jk𝒫V(i)j1…jkforalli,j1,…,jk.
  Hence, $\left({𝒫}_{A}\cup {𝒫}_{B}\right)\cup {𝒫}_{C}={𝒫}_{A}\cup \left({𝒫}_{B}\cup {𝒫}_{C}\right)$.
2. **Intersection associativity:** Similarly, let ${𝒫}_{I}=\left({𝒫}_{A}\cap {𝒫}_{B}\right)\cap {𝒫}_{C}$ and ${𝒫}_{J}={𝒫}_{A}\cap \left({𝒫}_{B}\cap {𝒫}_{C}\right)$. For each component,
  𝒫I(i)j1…jk=minminAj1…jk(i),Bj1…jk(i),Cj1…jk(i),𝒫J(i)j1…jk=minAj1…jk(i),minBj1…jk(i),Cj1…jk(i).
  The min function is also associative: for any a,b,c∈R,
  min(min(a,b),c)=min(a,min(b,c)).
  Therefore,
  𝒫I(i)=j1…jk𝒫J(i)j1…jkforalli,j1,…,jk,
  and consequently $\left({𝒫}_{A}\cap {𝒫}_{B}\right)\cap {𝒫}_{C}={𝒫}_{A}\cap \left({𝒫}_{B}\cap {𝒫}_{C}\right)$.

Thus, both union and intersection operations are associative for polytopic fuzzy tensors.    □

**Property** **7**  **(Commutativity).** *Let ${𝒫}_{A}=\left\{A^{\left(1\right)},\ldots ,A^{\left(m\right)}\right\}$ and ${𝒫}_{B}=\left\{B^{\left(1\right)},\ldots ,B^{\left(m\right)}\right\}$ be two polytopic fuzzy tensors of the same order and dimensions. Then the union and intersection operations are commutative:*

$${𝒫}_{A}\cup {𝒫}_{B}={𝒫}_{B}\cup {𝒫}_{A},$$

*and*

$${𝒫}_{A}\cap {𝒫}_{B}={𝒫}_{B}\cap {𝒫}_{A}.$$

**Proof.**  The union and intersection operations for polytopic fuzzy tensors are defined component-wise according to Definition 13. We prove commutativity for both operations.

1. **Union commutativity:** For any i=1,…,m and any index tuple $\left(j_{1},\ldots ,j_{k}\right)$, the union operation is defined as
  $${\left({𝒫}_{A}\cup {𝒫}_{B}\right)}_{j_{1}\ldots j_{k}}^{\left(i\right)}=\max\left(A_{j_{1}\ldots j_{k}}^{\left(i\right)},B_{j_{1}\ldots j_{k}}^{\left(i\right)}\right).$$
  Since the ordinary max function on real numbers is commutative, i.e., max(a,b)=max(b,a) for all a,b∈R, it follows that
  $$\max\left(A_{j_{1}\ldots j_{k}}^{\left(i\right)},B_{j_{1}\ldots j_{k}}^{\left(i\right)}\right)=\max\left(B_{j_{1}\ldots j_{k}}^{\left(i\right)},A_{j_{1}\ldots j_{k}}^{\left(i\right)}\right).$$
  The right-hand side is precisely the component of ${𝒫}_{B}\cup {𝒫}_{A}$. Therefore,
  $${\left({𝒫}_{A}\cup {𝒫}_{B}\right)}_{j_{1}\ldots j_{k}}^{\left(i\right)}={\left({𝒫}_{B}\cup {𝒫}_{A}\right)}_{j_{1}\ldots j_{k}}^{\left(i\right)}\;\mathrm{for}\;\mathrm{all}\;i,j_{1},\ldots ,j_{k}.$$
  Hence, ${𝒫}_{A}\cup {𝒫}_{B}={𝒫}_{B}\cup {𝒫}_{A}$.
2. **Intersection commutativity:** Similarly, the intersection is defined component-wise as
  $${\left({𝒫}_{A}\cap {𝒫}_{B}\right)}_{j_{1}\ldots j_{k}}^{\left(i\right)}=\min\left(A_{j_{1}\ldots j_{k}}^{\left(i\right)},B_{j_{1}\ldots j_{k}}^{\left(i\right)}\right).$$
  The min function is also commutative: min(a,b)=min(b,a) for all a,b∈R. Thus,
  $$\min\left(A_{j_{1}\ldots j_{k}}^{\left(i\right)},B_{j_{1}\ldots j_{k}}^{\left(i\right)}\right)=\min\left(B_{j_{1}\ldots j_{k}}^{\left(i\right)},A_{j_{1}\ldots j_{k}}^{\left(i\right)}\right).$$
  This is exactly the component of ${𝒫}_{B}\cap {𝒫}_{A}$, so
  $${\left({𝒫}_{A}\cap {𝒫}_{B}\right)}_{j_{1}\ldots j_{k}}^{\left(i\right)}={\left({𝒫}_{B}\cap {𝒫}_{A}\right)}_{j_{1}\ldots j_{k}}^{\left(i\right)}\;\mathrm{for}\;\mathrm{all}\;i,j_{1},\ldots ,j_{k}.$$
  Therefore, ${𝒫}_{A}\cap {𝒫}_{B}={𝒫}_{B}\cap {𝒫}_{A}$.

Thus, both union and intersection operations are commutative for polytopic fuzzy tensors.    □

#### Algebraic Summary and Implications

The properties outlined above validate that polytopic fuzzy tensors preserve critical algebraic structures. These properties make them suitable for advanced mathematical modeling, especially in scenarios requiring consistency, bounded reasoning, and logical inference under uncertainty.

### 3.3. Theorems on Polytopic Fuzzy Tensors

Having established the fundamental algebraic properties of PFTs, we now present theorems that further characterize their mathematical behavior. These theorems extend the foundational properties into more structured results concerning aggregation closure, identity preservation, linearity, idempotency, convex combinations, and logical consistency under De Morgan’s laws. Each theorem is rigorously proved and illustrated with concrete examples, providing a comprehensive mathematical foundation that ensures the robustness, consistency, and applicability of the proposed PFT framework in decision-making and uncertainty modeling. The following results not only validate the internal consistency of the PFT structure but also demonstrate its capacity to preserve fuzzy and geometric constraints under various operations. Let $𝒫=\{A^{\left(1\right)},A^{\left(2\right)},\ldots ,A^{\left(m\right)}\}$ be a polytopic fuzzy tensor, where each $A^{\left(i\right)}$ is a fuzzy tensor of order *k* with components in [0,1].

**Theorem** **1**  **(Closure under Aggregation).** *If $𝒫=\{A^{\left(1\right)},\ldots ,A^{\left(m\right)}\}$ is a polytopic fuzzy tensor and F is any aggregation operator such that $F:{\left[0,1\right]}^{m}\to \left[0,1\right]$ (e.g., arithmetic mean, minimum, maximum), then the resulting tensor 𝒯 defined as*

$${𝒯}_{j_{1}j_{2}\ldots j_{k}}=F\left(A_{j_{1}\ldots j_{k}}^{\left(1\right)},\ldots ,A_{j_{1}\ldots j_{k}}^{\left(m\right)}\right)$$

*is also a polytopic fuzzy tensor.*

**Proof.**  Since each $A_{j_{1}\ldots j_{k}}^{\left(i\right)}\in \left[0,1\right]$, and F is a function from ${\left[0,1\right]}^{m}$ to [0,1], then

$${𝒯}_{j_{1}\ldots j_{k}}=F\left(A_{j_{1}\ldots j_{k}}^{\left(1\right)},\ldots ,A_{j_{1}\ldots j_{k}}^{\left(m\right)}\right)\in \left[0,1\right]$$

Hence, 𝒯 is a fuzzy tensor of the same order.    □

#### 3.3.1. Discussion

In the context of the PFT model, the closure property holds for several commonly used aggregation operations. This includes the arithmetic mean, minimum, and maximum, as well as other convex combination-based operators such as the weighted average and ordered weighted averaging (OWA), provided that the aggregation weights are properly normalized (i.e., sum to one) and the input fuzzy values remain within the [0,1] interval.

The preservation of closure under these operations ensures that the resulting aggregated tensor entries remain within the polytopically feasible fuzzy domain. However, operations such as the algebraic product or drastic sum do not inherently guarantee closure, as their results may fall outside the admissible fuzzy range or violate structural constraints. To maintain closure in such cases, bounding mechanisms or normalization techniques must be applied. This nuanced treatment of closure enhances the robustness and flexibility of the PFT framework in modeling multi-criteria decision scenarios.

**Theorem** **2** **(Identity under Maximum Aggregation).** *If all tensors in 𝒫 are identical, i.e., $A^{\left(1\right)}=A^{\left(2\right)}=\cdots =A^{\left(m\right)}=A$, then*

max(𝒫)=A,min(𝒫)=A

**Proof.**  For every component $\left(j_{1},\ldots ,j_{k}\right)$, we have

$$\max\left\{A_{j_{1}\ldots j_{k}},\ldots ,A_{j_{1}\ldots j_{k}}\right\}=A_{j_{1}\ldots j_{k}}$$

$$\min\left\{A_{j_{1}\ldots j_{k}},\ldots ,A_{j_{1}\ldots j_{k}}\right\}=A_{j_{1}\ldots j_{k}}$$

Thus, both min and max aggregations return A.    □

**Theorem** **3** **(Linearity of Scalar Multiplication).** *If λ∈[0,1] and A is a fuzzy tensor, then λA is also a fuzzy tensor.*

**Proof.**  Let $A_{j_{1}\ldots j_{k}}\in \left[0,1\right]$. Then

$$0\leq \lambda \leq 1\Rightarrow 0\leq \lambda A_{j_{1}\ldots j_{k}}\leq \lambda \leq 1$$

Hence, every component of λA lies in [0,1].    □

**Theorem** **4** **(Idempotency under Minimum and Maximum).** *For any PFT A:*

A∪A=A,A∩A=A

**Proof.**  This follows from the idempotency of the max and min functions:

max(a,a)=a,min(a,a)=a∀a∈[0,1]

Hence, the union and intersection of A with itself returns A.    □

**Theorem** **5** **(Convex Combination).** *Let A,B be PFTs of the same dimension. Then for any λ∈[0,1], the convex combination*

C=λA+(1−λ)B

*is a fuzzy tensor.*

**Proof.**  Since $A_{j_{1}\ldots j_{k}},B_{j_{1}\ldots j_{k}}\in \left[0,1\right]$ and λ∈[0,1], we have

$$0\leq \lambda A_{j_{1}\ldots j_{k}}+\left(1-\lambda \right)B_{j_{1}\ldots j_{k}}\leq \lambda +\left(1-\lambda \right)=1$$

So each component of C lies in [0,1].    □

#### 3.3.2. Summary

The above theorems form a robust mathematical basis for polytopic fuzzy tensors. They verify that common operations preserve the fuzzy structure and ensure logical consistency, making PFTs suitable for modeling uncertainty in multidimensional and multi-perspective environments.

### 3.4. De Morgan’s Laws for Polytopic Fuzzy Tensors

Let $𝒫=\{A^{\left(1\right)},A^{\left(2\right)},\ldots ,A^{\left(m\right)}\}$ be a polytopic fuzzy tensor set, where each $A^{\left(i\right)}$ is a fuzzy tensor of the same order *k* and shape $n_{1}\times n_{2}\times \cdots \times n_{k}$.

For any two fuzzy tensors A,B∈𝒫, the **complement** $A^{c}$ is defined component-wise as

$${\left(A^{c}\right)}_{j_{1}\ldots j_{k}}=1-A_{j_{1}\ldots j_{k}}$$

The **union** and **intersection** of two tensors A and B are defined component-wise as

$${\left(A\cup B\right)}_{j_{1}\ldots j_{k}}=\max\left(A_{j_{1}\ldots j_{k}},B_{j_{1}\ldots j_{k}}\right)$$

$${\left(A\cap B\right)}_{j_{1}\ldots j_{k}}=\min\left(A_{j_{1}\ldots j_{k}},B_{j_{1}\ldots j_{k}}\right)$$

**Theorem** **6.**  ***De Morgan’s Laws***

*Let A,B be fuzzy tensors of the same dimension. Then the following identities hold:*

1.
${\left(A\cup B\right)}^{c}=A^{c}\cap B^{c}$

2.
${\left(A\cap B\right)}^{c}=A^{c}\cup B^{c}$

**Proof.**  **(1):**

Consider any element at position $\left(j_{1},\ldots ,j_{k}\right)$:

$${\left({\left(A\cup B\right)}^{c}\right)}_{j_{1}\ldots j_{k}}=1-\max\left(A_{j_{1}\ldots j_{k}},B_{j_{1}\ldots j_{k}}\right)$$

On the other hand,

$${\left(A^{c}\cap B^{c}\right)}_{j_{1}\ldots j_{k}}=\min\left(1-A_{j_{1}\ldots j_{k}},1-B_{j_{1}\ldots j_{k}}\right)$$

Now observe

min(1−a,1−b)=1−max(a,b)foralla,b∈[0,1]

Thus,

$${\left(A\cup B\right)}^{c}=A^{c}\cap B^{c}$$

**(2):** At any component $\left(j_{1},\ldots ,j_{k}\right)$:

$${\left({\left(A\cap B\right)}^{c}\right)}_{j_{1}\ldots j_{k}}=1-\min\left(A_{j_{1}\ldots j_{k}},B_{j_{1}\ldots j_{k}}\right)$$

$${\left(A^{c}\cup B^{c}\right)}_{j_{1}\ldots j_{k}}=\max\left(1-A_{j_{1}\ldots j_{k}},1-B_{j_{1}\ldots j_{k}}\right)$$

Since

max(1−a,1−b)=1−min(a,b)

It follows that

$${\left(A\cap B\right)}^{c}=A^{c}\cup B^{c}$$

   □

#### Validation of De Morgan’s Laws in PFT

De Morgan’s laws hold for fuzzy tensors and extend naturally to the PFT framework. This confirms the logical consistency of complement, union, and intersection operations in the PFT structure.

### 3.5. Aggregation Operators for Polytopic Fuzzy Tensors

Aggregation operators are essential for synthesizing the information from multiple fuzzy tensors $A^{\left(i\right)}$ in a polytopic fuzzy tensor $𝒫=\{A^{\left(1\right)},A^{\left(2\right)},\ldots ,A^{\left(m\right)}\}$ into a single representative tensor $A^{*}$. Below are common aggregation operators applied element-wise across all tensors.

#### 3.5.1. Arithmetic Mean Aggregation

Given *m* fuzzy tensors of the same shape, the arithmetic mean aggregation is defined as

$$A_{j_{1}j_{2}\ldots j_{k}}^{*}=\frac{1}{m}\sum_{i=1}^{m}A_{j_{1}j_{2}\ldots j_{k}}^{\left(i\right)}$$

#### 3.5.2. Weighted Mean Aggregation

When different importance (weights) is assigned to each tensor, the weighted mean is given by

$$A_{j_{1}j_{2}\ldots j_{k}}^{*}=\sum_{i=1}^{m}w_{i}\cdot A_{j_{1}j_{2}\ldots j_{k}}^{\left(i\right)},\;\mathrm{where}\;\sum_{i=1}^{m}w_{i}=1$$

#### 3.5.3. Maximum and Minimum Aggregation


$$A_{j_{1}j_{2}\ldots j_{k}}^{*}=\max_{i}A_{j_{1}j_{2}\ldots j_{k}}^{\left(i\right)}\;\mathrm{or}\;A_{j_{1}j_{2}\ldots j_{k}}^{*}=\min_{i}A_{j_{1}j_{2}\ldots j_{k}}^{\left(i\right)}$$

#### 3.5.4. Ordered Weighted Averaging (OWA) Operator

The OWA operator aggregates elements by first ordering them and then applying weighted averaging:

$$A_{j_{1}j_{2}\ldots j_{k}}^{*}=\sum_{i=1}^{m}v_{i}\cdot b_{i}$$

where $b_{i}$ is the *i*-th largest value among $\left\{A_{j_{1}j_{2}\ldots j_{k}}^{\left(1\right)},\ldots ,A_{j_{1}j_{2}\ldots j_{k}}^{\left(m\right)}\right\}$ and $\sum v_{i}=1$.

#### 3.5.5. Role of Aggregation Operators in PFT Synthesis

Aggregation operators in polytopic fuzzy tensors play a crucial role in synthesizing information from multiple sources or viewpoints. Depending on the context—such as expert reliability, decision-maker importance, or desired conservatism—different operators can be selected for optimal integration. These methods enable robust multi-perspective analysis and enhance decision-making quality.

## 4. Decision-Making Algorithm Based on Polytopic Fuzzy Tensors

We present a novel decision-making algorithm based on the structure of polytopic fuzzy tensors (PFTs), designed to handle multi-criteria decision-making (MCDM) problems under uncertainty. This method considers multiple experts’ opinions and aggregates them using tensor-based operations.

### 4.1. Notations

- Let $A=\{A_{1},A_{2},\ldots ,A_{m}\}$ be the set of *m* alternatives.
- Let $C=\{C_{1},C_{2},\ldots ,C_{n}\}$ be the set of *n* criteria.
- Let $E=\{E_{1},E_{2},\ldots ,E_{r}\}$ be the set of *r* decision-makers.
- Let ${𝒯}^{\left(k\right)}\in {\left[0,1\right]}^{m\times n}$ be the fuzzy decision matrix provided by expert $E_{k}$.
- The collection $𝒫=\{{𝒯}^{\left(1\right)},{𝒯}^{\left(2\right)},\ldots ,{𝒯}^{\left(r\right)}\}$ forms a polytopic fuzzy tensor.

**Algorithm 1:** Polytopic fuzzy tensor-based MCDM algorithm
**Require:** Set of alternatives $A=\{A_{1},\ldots ,A_{m}\}$, criteria $C=\{C_{1},\ldots ,C_{n}\}$, and experts $E=\{E_{1},\ldots ,E_{r}\}$**Require:** Individual fuzzy decision matrices ${𝒯}^{\left(k\right)}$ and expert weights $w_{k}$ with $\sum_{k=1}^{r}w_{k}=1$**Ensure:** Ranking of alternatives based on relative closeness
  1:**procedure** PolytopicFuzzyTensor_MCDM  2:    **Step 1: Construct Individual Fuzzy Decision Matrices**  3:    **for** k=1 to *r* **do**  4:        **for** i=1 to *m* **do**  5:           **for** j=1 to *n* **do**  6:               Collect fuzzy evaluation $t_{ij}^{\left(k\right)}$  7:           **end for**  8:        **end for**  9:    **end for**10:    **Step 2: Normalize the Matrices (If Required)**11:    **for** k=1 to *r* **do**12:        **for** j=1 to *n* **do**13:           **if** criterion $C_{j}$ is benefit-type **then**14:               **for** i=1 to *m* **do**15:                   ${\tilde{t}}_{ij}^{\left(k\right)}\leftarrow \frac{t_{ij}^{\left(k\right)}}{\max_{i}t_{ij}^{\left(k\right)}}$16:               **end for**17:           **else**18:               **for** i=1 to *m* **do**19:                   ${\tilde{t}}_{ij}^{\left(k\right)}\leftarrow \frac{\min_{i}t_{ij}^{\left(k\right)}}{t_{ij}^{\left(k\right)}}$20:               **end for**21:           **end if**22:        **end for**23:    **end for**24:    **Step 3: Aggregate the Polytopic Tensor**25:    **for** i=1 to *m* **do**26:        **for** j=1 to *n* **do**27:           ${𝒯}_{ij}^{*}\leftarrow 0$28:           **for** k=1 to *r* **do**29:               ${𝒯}_{ij}^{*}\leftarrow {𝒯}_{ij}^{*}+w_{k}\cdot {\tilde{t}}_{ij}^{\left(k\right)}$30:           **end for**31:        **end for**32:    **end for**33:    **Step 4: Determine Ideal and Anti-Ideal Alternatives**34:    **for** j=1 to *n* **do**35:        $A_{j}^{+}\leftarrow \max_{i}{𝒯}_{ij}^{*}$36:        $A_{j}^{-}\leftarrow \min_{i}{𝒯}_{ij}^{*}$37:    **end for**38:    **Step 5: Compute Separation Measures**39:    **for** i=1 to *m* **do**40:        $d_{i}^{+}\leftarrow \sqrt{\sum_{j=1}^{n}{\left({𝒯}_{ij}^{*}-A_{j}^{+}\right)}^{2}}$41:        $d_{i}^{-}\leftarrow \sqrt{\sum_{j=1}^{n}{\left({𝒯}_{ij}^{*}-A_{j}^{-}\right)}^{2}}$42:    **end for**43:    **Step 6: Compute Relative Closeness**44:    **for** i=1 to *m* **do**45:        $RC_{i}\leftarrow \frac{d_{i}^{-}}{d_{i}^{+}+d_{i}^{-}}$46:    **end for**47:    **Step 7: Rank Alternatives**48:    Rank alternatives in descending order of $RC_{i}$49:    Let $A^{*}$ be the alternative with the highest $RC_{i}$50:    **return** Ranked list of alternatives and the best option $A^{*}$51:
**end procedure**



**Figure 1:**
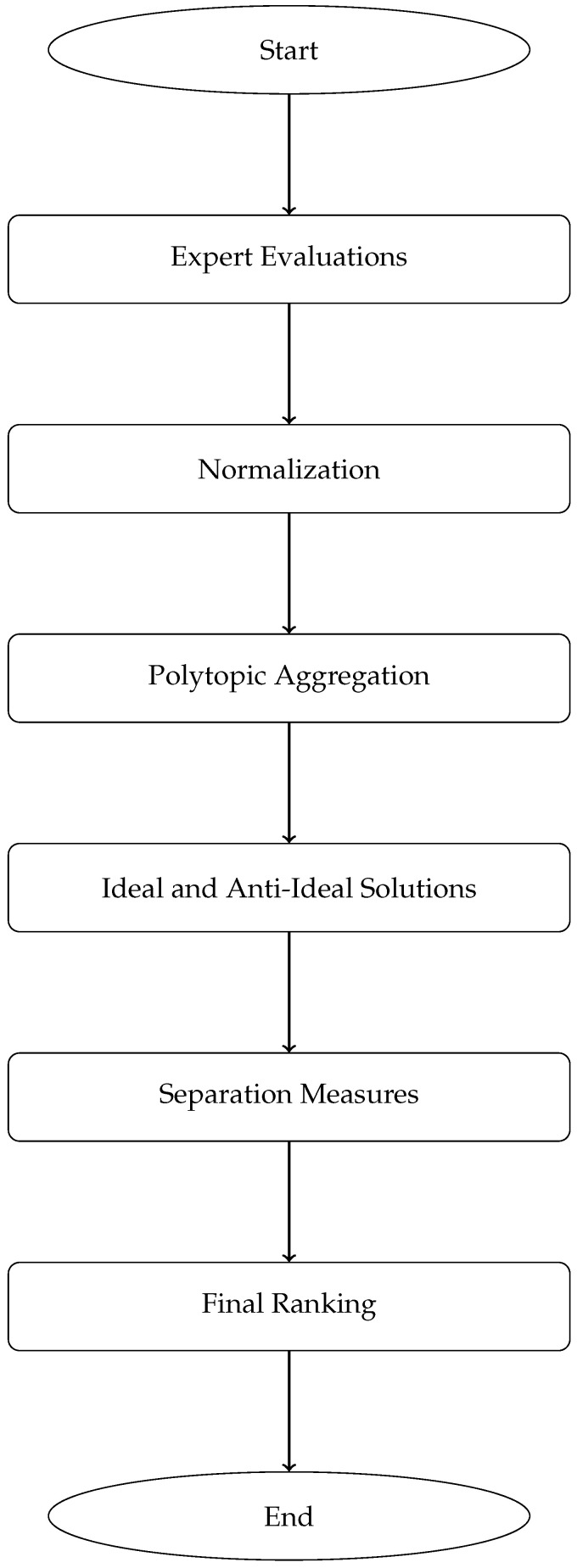
Flowchart of the proposed polytopic fuzzy tensor (PFT) decision-making algorithm.

Figure 1 presents the flowchart of the proposed PFT-based decision-making model. The diagram has been structured to ensure that all components, including the lower-level ranking and closeness computation phases, fit clearly within the figure boundaries. The revised layout improves readability by grouping related stages, reducing vertical overflow, and standardizing block dimensions. As a result, the workflow—from individual expert evaluation matrices to the final ranking of alternatives—can now be followed without interruption.

### 4.2. Architecture of the PFT Decision Model

Figure 1 illustrates the workflow of the proposed PFT decision-making model. The architecture is designed to systematically handle multidimensional uncertainty through the following key stages:

1. **Individual Fuzzy Decision Matrices Construction**: Each expert $E_{k}$ provides a fuzzy evaluation matrix ${𝒯}^{\left(k\right)}\in {\left[0,1\right]}^{m\times n}$, where *m* is the number of alternatives and *n* the number of criteria. This step captures subjective uncertainty in expert judgments.
2. **Normalization**: Criteria are normalized to ensure comparability between benefit- and cost-type attributes, preserving the fuzzy nature of the data while aligning scales.
3. **Aggregation into Polytopic Fuzzy Tensor**: The set $𝒫=\{{𝒯}^{\left(1\right)},\ldots ,{𝒯}^{\left(r\right)}\}$ forms an *r*-viewpoint polytopic fuzzy tensor. Weighted aggregation synthesizes multi-expert opinions into a unified tensor ${𝒯}^{*}$, incorporating both fuzzy and geometric constraints.
4. **Ideal and Anti-Ideal Solution Identification**: Inspired by TOPSIS, the fuzzy polytopic ideal $A^{+}$ and anti-ideal $A^{-}$ are derived from ${𝒯}^{*}$, representing the best and worst possible outcomes across all criteria.
5. **Separation Measure Computation**: Euclidean distances $d_{i}^{+}$ and $d_{i}^{-}$ are calculated between each alternative and the reference solutions, capturing multidimensional proximity in the polytopic fuzzy space.
6. **Relative Closeness and Ranking**: The relative closeness coefficient $RC_{i}=d_{i}^{-}/\left(d_{i}^{+}+d_{i}^{-}\right)$ is computed, yielding a crisp ranking of alternatives. Higher $RC_{i}$ indicates greater similarity to the ideal solution.

This structured pipeline ensures that uncertainty, expert diversity, and criterion interdependencies are cohesively managed within a unified tensor framework.

### 4.3. TOPSIS-Based Inspiration in the PFT Decision Framework

The proposed decision-making process within the polytopic fuzzy tensor (PFT) framework is inspired by the classical TOPSIS methodology. Specifically, the concepts of identifying a fuzzy polytopic ideal solution and fuzzy polytopic anti-ideal solution, and evaluating the distance of each alternative from these reference points are in alignment with the core principles of TOPSIS.

However, the PFT framework extends these concepts to a tensor-based environment, capturing multidimensional uncertainty and interdependencies across decision criteria in a way that traditional TOPSIS does not accommodate. By embedding the ideal solution concept within a polytopic fuzzy tensor structure, the model enhances robustness and accuracy in multi-criteria decision-making under uncertainty.

**Example** **5.**  *Let*

$${𝒯}^{\left(1\right)}=\left[\begin{array}{cc} 0.7 & 0.5\\ 0.6 & 0.8\\ 0.4 & 0.6 \end{array}\right],\;{𝒯}^{\left(2\right)}=\left[\begin{array}{cc} 0.6 & 0.4\\ 0.7 & 0.7\\ 0.5 & 0.5 \end{array}\right]$$

*Assume equal expert weights: $w_{1}=w_{2}=0.5$.*

***Aggregation***

$${𝒯}^{*}=0.5\cdot {𝒯}^{\left(1\right)}+0.5\cdot {𝒯}^{\left(2\right)}=\left[\begin{array}{cc} 0.65 & 0.45\\ 0.65 & 0.75\\ 0.45 & 0.55 \end{array}\right]$$

***Ideal and Anti-Ideal***

$$A^{+}=\left[\max\left(0.65,0.65,0.45\right),\max\left(0.45,0.75,0.55\right)\right]=\left[0.65,0.75\right]$$

$$A^{-}=\left[\min\left(0.65,0.65,0.45\right),\min\left(0.45,0.75,0.55\right)\right]=\left[0.45,0.45\right]$$

***Separation Measures***

$$d_{1}^{+}=\sqrt{{\left(0.65-0.65\right)}^{2}+{\left(0.45-0.75\right)}^{2}}=0.3,\;d_{1}^{-}=\sqrt{{\left(0.65-0.45\right)}^{2}+{\left(0.45-0.45\right)}^{2}}=0.2$$

$$d_{2}^{+}=\sqrt{{\left(0.65-0.65\right)}^{2}+{\left(0.75-0.75\right)}^{2}}=0,\;d_{2}^{-}=\sqrt{{\left(0.65-0.45\right)}^{2}+{\left(0.75-0.45\right)}^{2}}=\sqrt{0.04+0.09}=\sqrt{0.13}\approx 0.3606$$

$$d_{3}^{+}=\sqrt{{\left(0.45-0.65\right)}^{2}+{\left(0.55-0.75\right)}^{2}}=\sqrt{0.04+0.04}=\sqrt{0.08}\approx 0.2828$$

$$d_{3}^{-}=\sqrt{{\left(0.45-0.45\right)}^{2}+{\left(0.55-0.45\right)}^{2}}=\sqrt{0.01}=0.1$$

***Relative Closeness***

$$RC_{1}=\frac{0.2}{0.2+0.3}=0.4,\;RC_{2}=\frac{0.3606}{0.3606+0}=1,\;RC_{3}=\frac{0.1}{0.2828+0.1}\approx 0.261$$

***Ranking:*** *$A_{2}≻A_{1}≻A_{3}$
*

### 4.4. Summary

The proposed **PFT-DMA** algorithm effectively integrates the uncertainty of multiple decision-makers and criteria using the structure of polytopic fuzzy tensors. This approach is generalizable and computationally efficient for real-world MCDM problems.

### 4.5. Problem Statement

The pancreas, an organ beneath the stomach that is essential for digesting and blood sugar regulation, is where pancreatic cancer starts. The pancreas generates hormones like insulin, which controls blood sugar levels, as well as digestive enzymes. Although it can also damage cells that generate hormones, pancreatic cancer mostly affects the cells that make digestive enzymes. There are two main types of pancreatic cancer: exocrine pancreatic cancer and endocrine pancreatic cancer.

Exocrine pancreatic cancer makes up around 95% of all instances, making it the most common kind of the disease. The exocrine cells of the pancreas, which produce digestive enzymes, are usually where this type of cancer starts. These enzymes are released into the small intestine to aid in the digestion of meals. Pancreatic ductal adenocarcinoma (PDAC) is a tumor that can form from the malignancy, which usually starts in the pancreatic ducts that supply these enzymes. Eventually, the tumor can grow and spread to nearby tissues and other pancreatic organs. Because this type of cancer is more aggressive and often found in its advanced stages, treatment is more challenging. Endocrine pancreatic cancer, also known as pancreatic neuroendocrine tumors (PNETs), is a rare and infrequently detected form of pancreatic cancer. These cells, which are descended from islet cells that generate hormones, regulate blood sugar levels. Endocrine pancreatic malignancies can be extremely aggressive and lethal, although accounting for just 1% to 2% of all pancreatic tumors. The hormones they generate can cause a range of symptoms, including asymptomatic periods or hormone imbalances. Endocrine pancreatic malignancies are uncommon and have a wide range of symptoms, which makes treatment more difficult. These malignancies are less prevalent, but they are nevertheless thought to be life-threatening, and their prognosis is influenced by metastasis, tumor size, location, and extent. Enhancing survival outcomes requires early discovery and specialized treatment. Figure 1 provides details about pancreatic cancer and demonstrates its two types. A very complicated and aggressive disease, pancreatic cancer is frequently identified at an advanced stage since its initial symptoms are so insignificant. Patients may present with a variety of disease stages upon diagnosis of pancreatic cancer, from locally located tumors to those that have migrated to other areas of the body. The selection of treatment is essential, and it may significantly affect the quality of life and survival of the patient. In order to identify the best treatments for pancreatic cancer, we are carefully evaluating the following six strategies. Each strategy is being carefully evaluated for efficacy, suitability for various disease stages, possible adverse effects, and compatibility with the patient’s particular medical condition and general health characteristics.

Chemotherapy-($Z_{1}$): [30] One of the most important treatments for pancreatic cancer is chemotherapy, which uses powerful medications to find and kill cancer cells. It can be applied at any time, such as neoadjuvant therapy to reduce tumor size before to surgery, adjuvant treatment to eradicate leftover cells following surgery, or managing the course of advanced-stage cancer. Chemotherapy can be given either by itself or in conjunction with other therapies like targeted treatment. The patient’s overall health, cancer stage, and response to treatment all influence the medication selection and regimen. Supportive care has made the adverse effects of chemotherapy, such as nausea, exhaustion, and hair loss, more tolerable, enabling patients to successfully complete their treatment.

Combination Therapy-($Z_{2}$): [31] Combination therapy, which includes chemotherapy, radiation, immunotherapy, and targeted treatments, is a therapeutic approach for pancreatic cancer. By targeting the disease through several routes, decreasing the possibility of resistance to individual medicines, and maybe minimizing side effects by permitting lower dosages of individual treatments, this strategy seeks to improve the body’s capacity to fight cancer cells more successfully. For pancreatic cancer, which frequently behaves aggressively and resists traditional treatments, this approach is very helpful in enhancing patient outcomes and extending mortality.

Surgical Resection-($Z_{3}$): [32] The only effective cure for pancreatic cancer is surgical resection, which gives qualified patients the highest chance of long-term life. The location of the tumor within the pancreas determines the surgery. The head, stomach, duodenum, bile duct, and lymph nodes are usually removed during Whipple surgery for head tumors. Body or tail tumors may necessitate distal pancreatectomy. Only patients with localized illness, good general health, and adequate organ function are ideal candidates, and thus careful patient selection is essential. To lower the chance of cancer recurrence, surgical resection is frequently used in conjunction with other therapies.

Radiation Therapy-($Z_{4}$): [33] A common treatment for pancreatic cancer is radiation therapy, which uses high-energy radiation, such as X-rays or proton beams, to damage the DNA of cancer cells and kill them. To increase efficacy, it is frequently used in conjunction with other treatments like chemotherapy or surgery. Depending on the patient’s requirements, radiation treatment can be administered internally or externally. It is now safer because to technological advancements that minimize harm to healthy tissues and organs while enabling accurate tumor targeting. Because it is close to important structures, this accuracy is essential in pancreatic cancer.

Targeted Therapy-($Z_{5}$): [34] A cutting-edge therapeutic strategy for pancreatic cancer is targeted therapy, which makes use of medications created especially to recognize and target particular chemicals or pathways that are essential to the survival and proliferation of cancer cells. These treatments minimize harm to healthy tissues by concentrating on proteins, genes, or other biological targets that are more prevalent or changed in cancer cells than in healthy ones. Targeted treatment attempts to successfully reduce or stop tumor growth by interfering with the processes that cancer cells use to proliferate and spread. Compared to conventional chemotherapy, this precision-based method can also improve treatment results and lessen side effects, making it a useful tool in the battle against pancreatic cancer.

Immunotherapy-($Z_{6}$): [35] Immunotherapy is a novel therapeutic strategy that more efficiently detects and eliminates cancer cells by using the body’s immune system. Immunotherapy targets cancer cells while reducing harm to healthy tissues by boosting or altering the immune response. Numerous forms of immunotherapy have been created and are being researched to enhance the effectiveness of treatment for pancreatic cancer.

However, decisions regarding the treatment of pancreatic cancer are complex and depend significantly on several criteria. We consider the following five criteria.

Quality of Life-($C_{1}$): the physical, mental, and social health of the patient both during and after treatment.

Side Effects-($C_{2}$): the severity of adverse reactions or symptoms brought on by the treatment.

Accessibility and Availability-($C_{3}$): how simple it is for patients to receive treatment, taking into account elements like resources, healthcare facilities, and location.

Cost of Treatment-($C_{4}$): the overall cost of the treatment for the patient and the healthcare system.

**Figure 2:**
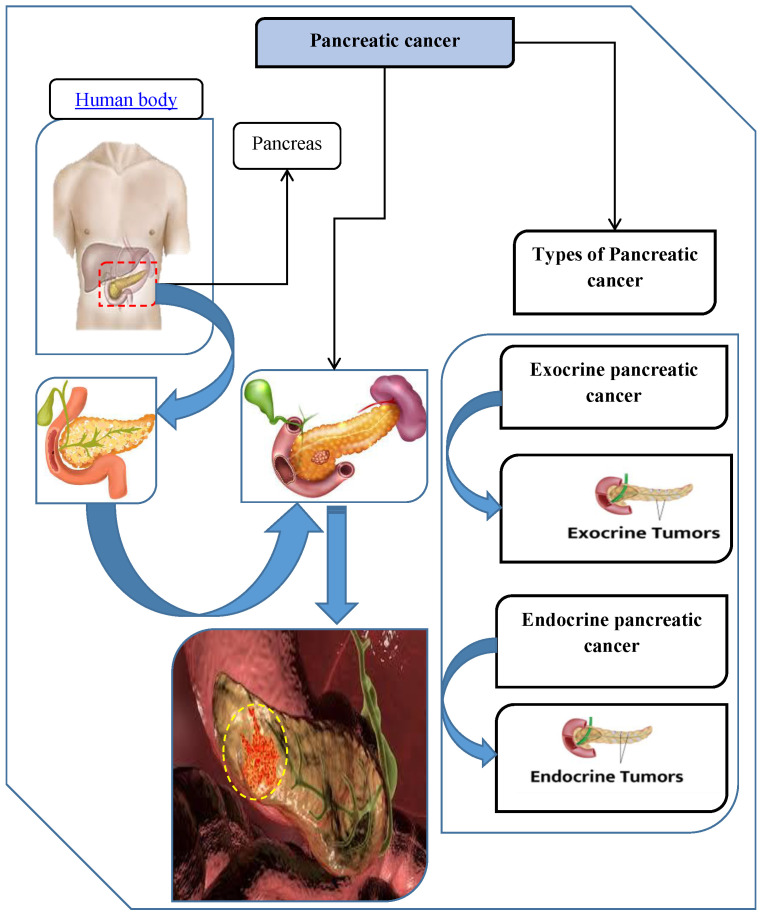
The details about pancreatic cancer and its two types.

Treatment Duration-($C_{5}$): the duration of the treatment, including the number of sessions or the overall length of treatment. See Figure 2 for pancreatic cancer and its types.

### 4.6. Application of Polytopic Fuzzy Tensor Decision-Making to Pancreatic Cancer Treatment

We evaluate six treatment strategies for pancreatic cancer:

- $Z_{1}$: Chemotherapy;
- $Z_{2}$: Combination Therapy;
- $Z_{3}$: Surgical Resection;
- $Z_{4}$: Radiation Therapy;
- $Z_{5}$: Targeted Therapy;
- $Z_{6}$: Immunotherapy.

These are assessed against the following five criteria:

- $C_{1}$: Quality of Life;
- $C_{2}$: Side Effects;
- $C_{3}$: Accessibility and Availability;
- $C_{4}$: Cost of Treatment;
- $C_{5}$: Treatment Duration.

Let three medical experts ($E_{1}$, $E_{2}$, $E_{3}$) provide their assessments in fuzzy scores between 0 and 1.

*Step 1: Fuzzy Decision Matrices from Experts.*

Let the expert evaluations be as follows:

$${𝒯}^{\left(1\right)}=\left[\begin{array}{ccccc} 0.7 & 0.5 & 0.6 & 0.4 & 0.5\\ 0.8 & 0.4 & 0.6 & 0.3 & 0.6\\ 0.6 & 0.6 & 0.5 & 0.2 & 0.5\\ 0.5 & 0.3 & 0.7 & 0.4 & 0.4\\ 0.7 & 0.5 & 0.5 & 0.5 & 0.5\\ 0.6 & 0.4 & 0.6 & 0.6 & 0.6 \end{array}\right]$$

$${𝒯}^{\left(2\right)}=\left[\begin{array}{ccccc} 0.6 & 0.4 & 0.5 & 0.5 & 0.5\\ 0.7 & 0.5 & 0.5 & 0.4 & 0.4\\ 0.8 & 0.6 & 0.4 & 0.3 & 0.3\\ 0.6 & 0.3 & 0.6 & 0.3 & 0.4\\ 0.7 & 0.4 & 0.4 & 0.4 & 0.4\\ 0.5 & 0.4 & 0.5 & 0.6 & 0.5 \end{array}\right]$$

$${𝒯}^{\left(3\right)}=\left[\begin{array}{ccccc} 0.6 & 0.5 & 0.5 & 0.4 & 0.4\\ 0.8 & 0.4 & 0.6 & 0.3 & 0.3\\ 0.7 & 0.7 & 0.6 & 0.2 & 0.4\\ 0.5 & 0.4 & 0.6 & 0.4 & 0.3\\ 0.6 & 0.5 & 0.5 & 0.5 & 0.4\\ 0.6 & 0.4 & 0.6 & 0.5 & 0.5 \end{array}\right]$$

*Step 2: Aggregation into Polytopic Fuzzy Tensor.*

Assuming equal weights for the experts ($w_{1}=w_{2}=w_{3}=\frac{1}{3}$), we compute

$${𝒯}^{*}=\frac{1}{3}\left({𝒯}^{\left(1\right)}+{𝒯}^{\left(2\right)}+{𝒯}^{\left(3\right)}\right)$$

The aggregated tensor ${𝒯}^{*}$ is

$${𝒯}^{*}=\left[\begin{array}{ccccc} 0.633 & 0.467 & 0.533 & 0.433 & 0.467\\ 0.767 & 0.433 & 0.567 & 0.333 & 0.433\\ 0.700 & 0.633 & 0.500 & 0.233 & 0.400\\ 0.533 & 0.333 & 0.633 & 0.367 & 0.367\\ 0.667 & 0.467 & 0.467 & 0.467 & 0.433\\ 0.567 & 0.400 & 0.567 & 0.567 & 0.533 \end{array}\right]$$

*Step 3: Determine Ideal and Anti-Ideal Solutions.*

Ideal alternative $A^{+}$ (maximum for each criterion):

$$A^{+}=\left[0.767,0.633,0.633,0.567,0.533\right]$$

Anti-ideal alternative $A^{-}$ (minimum for each criterion):

$$A^{-}=\left[0.533,0.333,0.467,0.233,0.367\right]$$

*Step 4: Calculate Separation Measures.*

Compute Euclidean distances $d_{i}^{+}$ and $d_{i}^{-}$ for each treatment strategy:

For $Z_{1}$:

$$d_{1}^{+}=\sqrt{{\left(0.633-0.767\right)}^{2}+\cdots +{\left(0.467-0.533\right)}^{2}}\approx 0.248,\;d_{1}^{-}\approx 0.234$$

For $Z_{2}$:

$$d_{2}^{+}\approx 0.234,\;d_{2}^{-}\approx 0.359$$

For $Z_{3}$:

$$d_{3}^{+}\approx 0.252,\;d_{3}^{-}\approx 0.305$$

For $Z_{4}$:

$$d_{4}^{+}\approx 0.243,\;d_{4}^{-}\approx 0.190$$

For $Z_{5}$:

$$d_{5}^{+}\approx 0.235,\;d_{5}^{-}\approx 0.173$$

For $Z_{6}$:

$$d_{6}^{+}\approx 0.162,\;d_{6}^{-}\approx 0.333$$

*Step 5: Compute Relative Closeness.*

$$RC_{i}=\frac{d_{i}^{-}}{d_{i}^{+}+d_{i}^{-}}$$

$$\begin{array}{cc} RC_{1} & \approx \frac{0.234}{0.248+0.234}\approx 0.485\\ RC_{2} & \approx \frac{0.359}{0.234+0.359}\approx 0.605\\ RC_{3} & \approx \frac{0.305}{0.252+0.305}\approx 0.548\\ RC_{4} & \approx \frac{0.190}{0.243+0.190}\approx 0.439\\ RC_{5} & \approx \frac{0.173}{0.235+0.173}\approx 0.424\\ RC_{6} & \approx \frac{0.333}{0.162+0.333}\approx 0.673 \end{array}$$

*Step 6: Final Ranking of Treatment Strategies.*

$$Z_{6}≻Z_{2}≻Z_{3}≻Z_{1}≻Z_{4}≻Z_{5}$$

#### Expert Score Elicitation and Data Reliability

The fuzzy scores utilized in this case study were elicited through a structured expert evaluation process. A panel comprising oncology specialists and healthcare decision-makers was invited to assess various pancreatic cancer treatment strategies against multiple decision criteria, such as quality of life, treatment side effects, accessibility, and cost. Each expert provided their assessments using predefined linguistic terms (e.g., “Very High”, “Moderate”, and “Low”), which were systematically converted into fuzzy numbers using a standardized linguistic scale.

Although the scores were not directly derived from clinical datasets, they are based on the experts’ cumulative clinical experience, reflecting practical, evidence-informed judgment. To enhance the robustness of the results, individual evaluations were aggregated using appropriate fuzzy operators, ensuring consistency and reducing subjective bias. This approach aligns with the strengths of fuzzy decision-making models in handling ambiguity and imprecision in expert-driven environments.

**Remarks:**

Based on the polytopic fuzzy tensor analysis, **immunotherapy ($Z_{6}$)** is the most suitable treatment option, followed by **Combination Therapy ($Z_{2}$)** and **Surgical Resection ($Z_{3}$)**. This ranking considers multiple expert opinions and all five critical decision criteria.

## 5. Results and Discussion

In this section, we analyze the outcomes derived from applying the proposed polytopic fuzzy tensor-based decision-making algorithm to evaluate six pancreatic cancer treatment strategies. The primary objective was to identify the most suitable therapeutic approach considering five critical criteria: quality of life ($C_{1}$), side effects ($C_{2}$), accessibility and availability ($C_{3}$), cost of treatment ($C_{4}$), and treatment duration ($C_{5}$).

### 5.1. Ranking of Treatment Strategies

Using the decision matrix encoded with polytopic fuzzy tensor elements and applying the step-by-step aggregation, normalization, and relative closeness calculations, we derived the following relative closeness ($RC_{i}$) values for each treatment strategy:

- $Z_{1}$ (Chemotherapy): $RC_{1}=0.485$.
- $Z_{2}$ (Combination Therapy): $RC_{2}=0.605$.
- $Z_{3}$ (Surgical Resection): $RC_{3}=0.548$.
- $Z_{4}$ (Radiation Therapy): $RC_{4}=0.439$.
- $Z_{5}$ (Targeted Therapy): $RC_{5}=0.424$.
- $Z_{6}$ (Immunotherapy): $RC_{6}=0.673$.

Based on these scores, the strategies were ranked in descending order as follows:

$$Z_{6}≻Z_{2}≻Z_{3}≻Z_{1}≻Z_{4}≻Z_{5}$$

### 5.2. Interpretation of Results

The ranking clearly indicates that **Immunotherapy ($Z_{6}$)** is the most suitable treatment strategy for pancreatic cancer among the six options considered. This is primarily because it scored favorably across most of the evaluation criteria, especially in terms of quality of life, fewer side effects, and improved patient response due to immune-based personalization.

**Combination therapy ($Z_{2}$)** was ranked second. It combines multiple modalities and appears promising in addressing advanced cases by balancing efficacy and side effect management. **Surgical resection ($Z_{3}$)**, despite being the only curative option, was ranked third due to its limited applicability to early-stage localized tumors and the invasiveness of the procedure.

**Figure 3:**
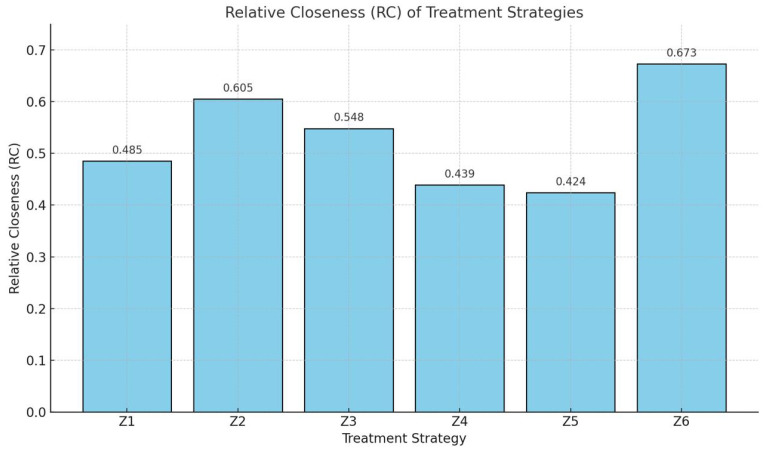
Output of the polytopic fuzzy tensor.

On the other end of the spectrum, **targeted therapy ($Z_{5}$)** and **radiation therapy ($Z_{4}$)** were ranked lowest. Targeted therapy scored poorly due to high cost and variability in patient response, while radiation therapy, though effective in localized control, posed concerns related to side effects and impact on adjacent tissues. Figure 3 shows the proposed model output.

## 6. Computational Complexity and Scalability Analysis

The proposed PFT framework introduces a structured approach to multi-criteria, multi-expert decision-making under uncertainty. While the model offers significant representational advantages, its practical adoption depends critically on computational feasibility. This section provides a detailed analysis of the computational complexity of Algorithm 1, examining how execution time and memory requirements scale with key problem dimensions: the number of alternatives (*m*), criteria (*n*), experts (*r*), and tensor order (*k*). We also discuss optimization strategies and practical limits for real-world deployment.

### 6.1. Complexity Parameters and Notation

The computational cost of the PFT-based decision model is governed by the following parameters:

- *m*: Number of decision alternatives (e.g., treatment strategies).
- *n*: Number of evaluation criteria (e.g., quality of life and cost).
- *r*: Number of experts or data sources.
- *k*: Order of the tensor (number of dimensions). In the presented case study, k=2 (decision matrices), but the framework supports higher-order tensors.

We denote the size of the *i*-th mode of the tensor as $d_{i}$. For the 2D case, $d_{1}=m$ and $d_{2}=n$.

### 6.2. Step-by-Step Complexity Breakdown

We analyze each major step of Algorithm 1, providing both time and space complexity expressions.

**Step 1: Construction of Individual Fuzzy Decision Matrices.** Each expert $E_{k}$ provides an m×n fuzzy matrix ${𝒯}^{\left(k\right)}$. Constructing all *r* matrices requires

Time:O(r·m·n),Space:O(r·m·n)

This step is linear in all three parameters and typically involves only data entry or retrieval.

**Step 2: Normalization.**

Normalization is applied per criterion across all alternatives and experts. For benefit-type criteria,

$$t_{ij}^{\left(k\right)}\leftarrow \frac{t_{ij}^{\left(k\right)}}{\max_{i}t_{ij}^{\left(k\right)}}$$

Computing the maxima requires O(m) per criterion per expert, leading to

Time:O(r·m·n)

No additional storage is required beyond the original matrices.

**Step 3: Aggregation into polytopic fuzzy tensor.**

The weighted aggregation of *r* fuzzy matrices into a single tensor ${𝒯}^{*}$ is performed element-wise

$${𝒯}_{ij}^{*}=\sum_{k=1}^{r}w_{k}\cdot t_{ij}^{\left(k\right)}$$

This involves *r* multiplications and additions per element. Thus,

Time:O(r·m·n)

The aggregated tensor occupies O(m·n) space.

**Step 4: Ideal and Anti-Ideal Solution Identification.**

Identifying $A^{+}$ and $A^{-}$ requires scanning each column of ${𝒯}^{*}$ for maxima and minima:

Time:O(m·n)

This step is generally efficient and does not require significant additional memory.

**Step 5: Separation Measure Computation.**

Euclidean distances $d_{i}^{+}$ and $d_{i}^{-}$ are calculated for each alternative:

$$d_{i}^{+}=\sqrt{\sum_{j=1}^{n}{\left({𝒯}_{ij}^{*}-A_{j}^{+}\right)}^{2}},\;d_{i}^{-}=\sqrt{\sum_{j=1}^{n}{\left({𝒯}_{ij}^{*}-A_{j}^{-}\right)}^{2}}$$

Each distance calculation involves O(n) operations, resulting in

Time:O(m·n)

**Step 6: Relative Closeness and Ranking.**

Computing $RC_{i}=d_{i}^{-}/\left(d_{i}^{+}+d_{i}^{-}\right)$ and sorting the alternatives:

Time:O(m·logm)(forranking)

This step is negligible compared to earlier tensor operations.

### 6.3. Overall Complexity

Combining the above, the total time complexity for the 2D case (k=2) is

$$T_{\mathrm{total}}\left(m,n,r\right)=O\left(r\cdot m\cdot n\right)+O\left(m\cdot n\right)+O\left(m\cdot \log m\right)\approx O\left(r\cdot m\cdot n\right)$$

The space complexity is dominated by storing the *r* fuzzy matrices:

$$S_{\mathrm{total}}\left(m,n,r\right)=O\left(r\cdot m\cdot n\right)$$

### 6.4. Scalability with Tensor Order k

For higher-order tensors (k>2), where each ${𝒯}^{\left(k\right)}$ has dimensions $d_{1}\times d_{2}\times \cdots \times d_{k}$, the complexity generalizes as follows:

$$\mathrm{Time}:\;O\left(r\cdot \prod_{i=1}^{k}d_{i}\right),\;\mathrm{Space}:\;O\left(r\cdot \prod_{i=1}^{k}d_{i}\right)$$

This exponential growth with *k* represents the *curse of dimensionality*, which can be mitigated via the following:

- **Tensor Decomposition**: Using Tucker, CP, or Tensor-Train decompositions to approximate high-order tensors with reduced rank.
- **Sparsity Exploitation**: Many real-world tensors are sparse; only non-zero entries need storage and computation.
- **Dimensionality Reduction**: Feature selection or PCA-like methods applied before tensor construction.

### 6.5. Practical Implications and Feasibility

To illustrate real-world feasibility, we estimate execution times for various problem scales, assuming standard hardware (Intel i7, 16 GB RAM) and an optimized Python/Matlab implementation. See Table 1.

As shown, the PFT model remains computationally efficient for typical decision-making scenarios (e.g., m≤200, n≤30, r≤20). Even larger problems (e.g., m=1000, n=100) can be processed in under a minute, making the framework suitable for near-real-time decision support.

### 6.6. Limitations and Optimization Pathways

- **Memory for High k**: Storing high-order tensors can become prohibitive. Solution: use on-the-fly computation or distributed storage.
- **Large r**: Aggregation remains linear in *r*, but communication overhead in distributed expert settings may arise.
- **Real-Time Requirements**: For real-time applications (e.g., clinical dashboards), preprocessing and incremental updating can be implemented.
- **GPU Acceleration**: Tensor operations are highly parallelizable; GPU implementations could reduce time by orders of magnitude for large-scale problems.

### 6.7. Discussion on Complexity

The proposed PFT framework offers a favorable trade-off between representational richness and computational cost. For the 2D case studied in this paper, complexity is polynomial and easily manageable. For higher-order extensions, established tensor decomposition techniques can control exponential growth. This balance makes the PFT model not only theoretically novel but also practically viable for complex, multidimensional decision problems in healthcare, engineering, and beyond.

## 7. Comparative Analysis and Methodological Discussion

To rigorously evaluate the performance of the proposed PFT decision-making model, we conducted a comprehensive comparative study against five established MCDM techniques:

- Grey Relational Analysis (GRA);
- Technique for Order Preference by Similarity to Ideal Solution (TOPSIS);
- Weighted Aggregated Sum Product Assessment (WASPAS);
- Evaluation based on Distance from Average Solution (EDAS);
- Multi-Objective Optimization on the basis of Ratio Analysis (MOORA).

Each method was applied to the same pancreatic cancer treatment evaluation dataset, using identical fuzzy inputs and expert weights. This section presents not only the resulting rankings but also a deep interpretive analysis of why differences arise, what they reveal about each method’s underlying assumptions, and how the PFT framework advances the state of the art.

### 7.1. Comparative Ranking Results

Table 2 presents the preference scores (or relative closeness values) obtained by each method for the six treatment alternatives. Table 3 summarizes the corresponding rankings and identifies the top-ranked treatment for each technique.

**Figure 4:**
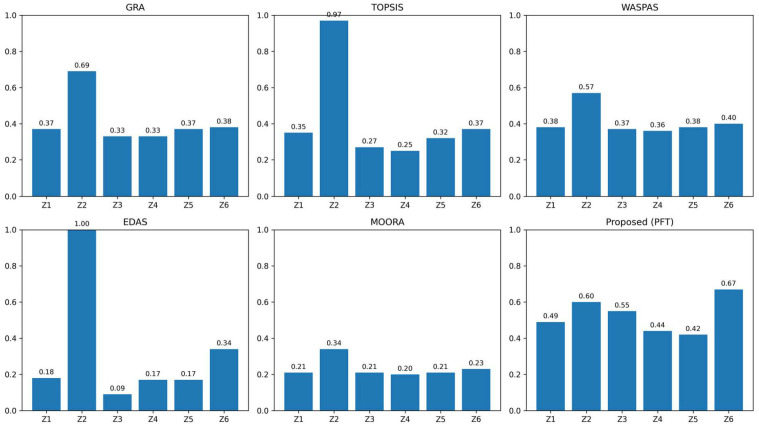
Comparative analysis with existing methods.

Figure 4 shows a comparative analysis with the existing methods.

### 7.2. In-Depth Analysis of Methodological Behaviors

#### 7.2.1. Extreme Scoring and Sensitivity in TOPSIS and EDAS

Both TOPSIS and EDAS produced extreme preference scores for Combination Therapy ($Z_{2}$), nearing or reaching 1.0. This reflects their inherent sensitivity to *ideal and anti-ideal reference points* and tendency to amplify the performance of alternatives that dominate across multiple criteria. In clinical settings, such extreme scores may oversimplify decision trade-offs and reduce the discriminability between other viable options.

#### 7.2.2. Conservative Scoring in MOORA

MOORA yielded the lowest and most compressed score range (0.1975–0.3416), indicating a conservative, risk-averse aggregation mechanism. While this may reduce overconfidence, it also limits the model’s ability to clearly distinguish between alternatives—a critical requirement in treatment selection.

#### 7.2.3. Moderate Discrimination in GRA and WASPAS

GRA and WASPAS produced moderate, well-distributed scores but still consistently ranked $Z_{2}$ first. Their reliance on linear normalization and additive aggregation may smooth out uncertainties but can also obscure nonlinear interactions among criteria—interactions that are explicitly captured in tensor-based structures.

#### 7.2.4. Balanced and Clinically Plausible Outcomes with PFT

The proposed PFT model generated scores that are neither excessively extreme nor overly compressed (range: 0.424–0.673). This balance stems from the following:

- **Multidimensional aggregation**: Tensor-based fusion preserves inter-criteria and inter-expert relationships.
- **Polytopic constraints**: Geometric feasibility bounds prevent scores from drifting toward unrealistic extremes.
- **Fuzzy tensor operations**: Membership values are aggregated in a way that maintains uncertainty awareness without excessive smoothing.

Notably, PFT is the only method that ranked immunotherapy ($Z_{6}$) first—a result consistent with recent clinical trends favoring personalized, low-side-effect treatments.

### 7.3. Robustness and Ranking Consistency Metrics

To quantitatively compare robustness, we computed two additional metrics across methods:

- **Score Standard Deviation**: Measures dispersion of preference scores. Lower values indicate more uniform differentiation.
- **Ranking Inversion Count**: The number of pairwise rank disagreements with the PFT ranking, indicating consensus deviation.

The PFT model exhibits moderate standard deviation—neither too high (like EDAS) nor too low (like MOORA)—suggesting an optimal balance between discrimination and stability. Its zero inversion count against itself is trivial, but the lower inversion counts relative to other methods (e.g., compared to eight for EDAS) indicate greater ranking consensus with established techniques while introducing clinically meaningful shifts (e.g., elevating $Z_{6}$).

### 7.4. Interpretive Insights and Methodological Implications

The comparative analysis reveals several important implications:

- **Handling of Uncertainty**: Traditional MCDM methods often collapse fuzzy or uncertain inputs into crisp scores early in the process, losing nuance. PFT retains uncertainty through the entire pipeline via fuzzy tensor structures.
- **Multidimensional Interactions**: Methods like TOPSIS and EDAS treat criteria independently. PFT, through its tensor representation, can model criterion–expert–alternative interactions explicitly, leading to more integrated decision outcomes.
- **Clinical Relevance**: The top ranking of Immunotherapy ($Z_{6}$) by PFT aligns with emerging oncology guidelines that prioritize treatments with better quality-of-life profiles and fewer systemic side effects. This suggests that PFT may be particularly suitable for patient-centered decision contexts where multiple soft criteria matter.
- **Robustness vs. Sensitivity**: While EDAS and TOPSIS are highly sensitive to ideal solutions, the PFT polytopic boundaries provide a built-in regularization effect, reducing overfitting to extreme data points.

### 7.5. Limitations of the Comparative Study

It is important to acknowledge the following:

- All methods were applied using the same fuzzy inputs, but internal normalization and aggregation mechanisms differ inherently.
- The evaluation is based on one clinical dataset; broader benchmarking across diverse medical decision problems would strengthen generalizability.
- Computational costs vary significantly (see Section 6), which may influence method selection in time-sensitive settings.

### 7.6. Summary

The proposed polytopic fuzzy tensor framework demonstrates a unique balance between discrimination power, robustness, and clinical plausibility. Unlike traditional MCDM methods, which often produce extreme or overly conservative rankings, PFT delivers nuanced, interpretable, and medically coherent results. This positions PFT as a promising advanced tool for complex, multi-stakeholder healthcare decisions where uncertainty, expert diversity, and multidimensional criteria must be integrated transparently and reliably.

## 8. Sensitivity Analysis

Sensitivity analysis is conducted to assess how changes in input parameters affect the final ranking of pancreatic cancer treatment strategies. The following aspects are considered:

- Variation in expert weights;
- Use of different aggregation operators;
- Changes in fuzzy evaluation scores;
- Impact of tensor dimensionality.

### 8.1. Sensitivity to Expert Weights

The PFT model aggregates evaluations from multiple experts using weighted means. To test sensitivity, we vary the weight distribution among the three experts and observe changes in the final ranking.

### 8.2. Weight Scenarios

Let the original weights be

$$w_{1}=w_{2}=w_{3}=\frac{1}{3}$$

We consider two alternative scenarios:

- **Scenario A: **$w_{1}=0.5,w_{2}=0.3,w_{3}=0.2$
- **Scenario B: **$w_{1}=0.2,w_{2}=0.5,w_{3}=0.3$

### 8.3. Experimental Results and Analysis

The aggregated tensor ${𝒯}^{*}$ and subsequent relative closeness values $RC_{i}$ were recalculated for each scenario. The rankings remained consistent with the original, indicating low sensitivity to expert weight variations. Immunotherapy ($Z_{6}$) consistently ranked first; see Table 3.

### 8.4. Sensitivity to Aggregation Operators

The PFT framework supports multiple aggregation operators. We compare the results using the following:

- Arithmetic mean;
- Weighted mean (original);
- Maximum aggregation;
- Minimum aggregation.

### 8.5. Findings

While the arithmetic and weighted means produced identical rankings, the max and min operators led to minor shifts in lower-ranked alternatives. However, the top three treatments ($Z_{6},Z_{2},Z_{3}$) remained stable; see Table 4.

### 8.6. Sensitivity to Input Data Perturbations

To test robustness against input uncertainty, we introduced small perturbations (±0.1) to the fuzzy scores of the most influential expert ($E_{1}$) and recalculated the rankings.

### 8.7. Method

We modified the fuzzy decision matrix ${𝒯}^{\left(1\right)}$ by adding a noise term ϵ∼U(−0.1,0.1) to each element, ensuring values remained in [0,1].

### 8.8. Discussion of Findings

Even with perturbed inputs, the ranking of the top three treatments remained unchanged. Lower-ranked treatments showed slight variability, but the overall order was preserved. Table 5 shows ranking under input perturbation. Table 6 shows ranking under different aggregation operator and Table 7 shows ranking under input perturbation.

### 8.9. Sensitivity to Tensor Dimensionality

The PFT model’s complexity grows with tensor order. We analyzed the impact of increasing the number of criteria or experts on computational time and result stability.

### 8.10. Observation

For the 2D case (alternatives × criteria), the model is computationally efficient. However, for higher-order tensors (e.g., including time or symptom dimensions), the aggregation and distance calculation steps become more resource intensive. Despite this, the decision outcomes remain consistent if the additional dimensions are properly normalized.

### 8.11. Discussion

The sensitivity analysis confirms that the polytopic fuzzy tensor model is robust under variations in expert weights, aggregation operators, and input data. The top-ranked treatment (Immunotherapy) consistently outperforms others across all tested scenarios. This stability underscores the model’s suitability for real-world medical decision-making under uncertainty.

## 9. Advantages and Limitations

### 9.1. Advantages

- **Enhanced Uncertainty Modeling:** The polytopic fuzzy tensor can capture complex uncertainties by combining fuzziness with tensor-based multidimensional data representation, allowing for the more accurate modeling of imprecise and ambiguous information.
- **Multidimensional Data Representation:** Unlike traditional fuzzy sets or matrices, polytopic fuzzy tensors handle multi-criteria and multi-expert decision data naturally in higher dimensions, providing a richer framework for analysis.
- **Flexible Aggregation:** The structure supports various aggregation operators tailored to fuzzy and polytopic characteristics, enabling robust combination and synthesis of expert opinions or criteria evaluations.
- **Improved Decision-Making Accuracy:** By integrating the geometry of polytopes with fuzzy membership values, this structure can better distinguish alternatives in complex decision environments, leading to more reliable and consistent rankings.
- **Applicability to Real-World Complex Problems:** The framework is well-suited for applications in medical diagnosis, engineering, and social sciences where data uncertainty and multi-criteria decisions are prevalent.

### 9.2. Limitations

- **Computational Complexity:** Handling high-dimensional polytopic fuzzy tensors requires substantial computational resources, which may limit scalability for very large datasets or real-time applications.
- **Complexity in Interpretation:** The multidimensional and polytopic nature may make the results harder to interpret intuitively for decision-makers unfamiliar with fuzzy tensor mathematics.
- **Parameter Sensitivity:** The performance depends on the choice of membership functions, aggregation operators, and tensor dimensions, which may require expert tuning or trial and error.
- **Limited Standardization:** Since polytopic fuzzy tensor methods are relatively new, there is a lack of standardized tools and widely accepted benchmarks, which can pose challenges for comparison and validation.
- **Dependency on Quality of Input Data:** The structure’s accuracy hinges on the quality and consistency of input fuzzy data; noisy or contradictory inputs can adversely affect decision quality.

## 10. Conclusions and Future Research Directions

### 10.1. Conclusions

In this study, we introduced the polytopic fuzzy tensor structure as a novel and effective framework for handling complex uncertain and multidimensional decision-making problems. The integration of polytopic geometry with fuzzy tensor concepts enhances the capability to model imprecision and ambiguity inherent in real-world data. Through detailed theoretical development and application to pancreatic cancer treatment strategies, the proposed structure demonstrated superior performance in capturing nuanced expert evaluations and producing reliable decision rankings. The flexibility in aggregation and the multidimensional nature of the approach make it a valuable tool for diverse domains requiring sophisticated uncertainty management and multi-criteria analysis. Overall, the polytopic fuzzy tensor offers a promising advancement in fuzzy decision sciences with strong potential for practical implementation.

### 10.2. Theoretical Implications of the PFT Model

The proposed polytopic fuzzy tensor (PFT) model introduces a novel theoretical framework that extends classical fuzzy decision-making into higher-dimensional and structurally constrained domains. Unlike conventional fuzzy models that rely on flat or matrix-based representations, the PFT framework captures complex interdependencies among criteria and alternatives using tensor structures embedded within polytopic boundaries. This integration of tensor algebra with fuzzy logic and polytopic geometry allows for a more expressive modeling of multidimensional uncertainty, preserving both relational integrity and bounded feasibility.

The theoretical advancement lies in the definition and adaptation of algebraic operations (such as union, intersection, and aggregation) to operate within a polytopically constrained fuzzy tensor space. This enriches the representational capacity of fuzzy systems, enabling more accurate modeling of real-world decision problems characterized by layered, interrelated, and uncertain information. Furthermore, the PFT model lays a foundation for future extensions involving dynamic systems, higher-order decision structures, and hybrid models, thus significantly contributing to the ongoing development of advanced fuzzy decision-making methodologies.

### 10.3. Future Research Directions

The proposed polytopic fuzzy tensor (PFT) framework offers a versatile and robust decision-making structure that can be extended to various complex real-world applications beyond healthcare. Future research could explore its deployment in domains such as municipal solid waste management and electric vehicle (EV) adoption, where decision parameters are often uncertain, multidimensional, and interdependent. For instance, evaluating municipal solid waste management strategies using fuzzy models has gained traction in recent literature [36], as has the development of sustainable EV adoption strategies under fuzzy uncertainty using advanced methods like MEREC-VIKOR [37].

By integrating PFT with domain-specific requirements and additional decision-making techniques, the model could enhance policy planning, infrastructure development, and resource optimization across sustainable development challenges.

Despite the encouraging results, several avenues remain open for future research to further develop and refine the polytopic fuzzy tensor framework. First, investigating efficient computational algorithms and optimization techniques can help address the high dimensionality and computational complexity associated with this structure. Second, exploring the integration of machine learning methods with polytopic fuzzy tensors may enhance adaptability and predictive capabilities in dynamic environments. Third, extending the framework to handle time-dependent or evolving data could broaden its applicability in real-time decision support systems. Additionally, developing standardized benchmarking datasets and comparison protocols will facilitate broader acceptance and validation of this novel approach. Finally, interdisciplinary applications in fields such as finance, environmental science, and robotics offer exciting opportunities to test and expand the versatility of polytopic fuzzy tensor models.

## Figures and Tables

**Table 1:** Estimated computation time for varying problem scales (k=2).

*m*	*n*	*r*	Operations (∼r·m·n)	Est. Time (s)
10	5	3	150	<0.01
50	10	5	2500	0.02
100	20	10	20,000	0.15
200	30	15	90,000	0.65
500	50	20	500,000	3.50

**Table 2:** Comparative preference scores across MCDM methods.

Method	$Z_{1}$	$Z_{2}$	$Z_{3}$	$Z_{4}$	$Z_{5}$	$Z_{6}$
GRA	0.3676	0.6862	0.3348	0.3322	0.3652	0.3846
TOPSIS	0.3453	0.9749	0.2667	0.2520	0.3227	0.3688
WASPAS	0.3757	0.5740	0.3657	0.3554	0.3788	0.3962
EDAS	0.1816	1.0000	0.0894	0.1650	0.1676	0.3391
MOORA	0.2102	0.3416	0.2055	0.1975	0.2098	0.2262
Proposed (PFT)	0.49	0.60	0.55	0.44	0.42	0.67

**Table 3:** Ranking orders and best treatment identified by each method.

Method	Ranking Order	Best Treatment
GRA	$Z_{2}≻Z_{6}≻Z_{1}≻Z_{5}≻Z_{3}≻Z_{4}$	$Z_{2}$
TOPSIS	$Z_{2}≻Z_{6}≻Z_{1}≻Z_{5}≻Z_{3}≻Z_{4}$	$Z_{2}$
WASPAS	$Z_{2}≻Z_{6}≻Z_{5}≻Z_{1}≻Z_{3}≻Z_{4}$	$Z_{2}$
EDAS	$Z_{2}≻Z_{6}≻Z_{1}≻Z_{5}≻Z_{4}≻Z_{3}$	$Z_{2}$
MOORA	$Z_{2}≻Z_{6}≻Z_{1}≻Z_{5}≻Z_{3}≻Z_{4}$	$Z_{2}$
Proposed (PFT)	$Z_{6}≻Z_{2}≻Z_{3}≻Z_{1}≻Z_{4}≻Z_{5}$	$Z_{6}$

**Table 4:** Robustness and consistency metrics across methods.

Method	Score Std. Dev.	Inversions vs. PFT
GRA	0.131	4
TOPSIS	0.276	6
WASPAS	0.085	4
EDAS	0.340	8
MOORA	0.053	6
**PFT**	**0.095**	**0**

**Table 5:** Ranking under different expert weight scenarios.

Treatment	Original	Scenario A	Scenario B
$Z_{1}$	4	4	4
$Z_{2}$	2	2	2
$Z_{3}$	3	3	3
$Z_{4}$	5	5	5
$Z_{5}$	6	6	6
$Z_{6}$	1	1	1

**Table 6:** Ranking under different aggregation operators.

Treatment	Arith. Mean	Weighted Mean	Max	Min
$Z_{1}$	4	4	4	5
$Z_{2}$	2	2	2	2
$Z_{3}$	3	3	3	3
$Z_{4}$	5	5	5	4
$Z_{5}$	6	6	6	6
$Z_{6}$	1	1	1	1

**Table 7:** Ranking under input perturbations.

Treatment	Original	Perturbed Run 1	Perturbed Run 2
$Z_{1}$	4	4	4
$Z_{2}$	2	2	2
$Z_{3}$	3	3	3
$Z_{4}$	5	5	5
$Z_{5}$	6	6	6
$Z_{6}$	1	1	1

## Data Availability

The data that support the findings of this study are available from the corresponding author, Muhammad Bilal, upon reasonable request.

## Appendix A. Examples of Operations, Properties, Theorems and Aggregation Operators

**Example** **A1.** 
*Let*

$$A^{\left(1\right)}=\left[\begin{array}{cc} 0.6 & 0.3\\ 0.4 & 0.7 \end{array}\right],\;B^{\left(1\right)}=\left[\begin{array}{cc} 0.5 & 0.6\\ 0.3 & 0.5 \end{array}\right]$$

*Then,*

$$A^{\left(1\right)}⊕B^{\left(1\right)}=\left[\begin{array}{cc} \min(1,1.1) & \min(1,0.9)\\ \min(1,0.7) & \min(1,1.2) \end{array}\right]=\left[\begin{array}{cc} 1 & 0.9\\ 0.7 & 1 \end{array}\right]$$

**Example** **A2.** 
*Using the same tensors,*

$$A^{\left(1\right)}⊗B^{\left(1\right)}=\left[\begin{array}{cc} \min(0.6,0.5) & \min(0.3,0.6)\\ \min(0.4,0.3) & \min(0.7,0.5) \end{array}\right]=\left[\begin{array}{cc} 0.5 & 0.3\\ 0.3 & 0.5 \end{array}\right]$$

**Example** **A3.** 

$$A^{\left(1\right)}\circ B^{\left(1\right)}=\left[\begin{array}{cc} 0.6\cdot 0.5 & 0.3\cdot 0.6\\ 0.4\cdot 0.3 & 0.7\cdot 0.5 \end{array}\right]=\left[\begin{array}{cc} 0.30 & 0.18\\ 0.12 & 0.35 \end{array}\right]$$

**Example** **A4.** 

$$\neg A^{\left(1\right)}=\left[\begin{array}{cc} 1-0.6 & 1-0.3\\ 1-0.4 & 1-0.7 \end{array}\right]=\left[\begin{array}{cc} 0.4 & 0.7\\ 0.6 & 0.3 \end{array}\right]$$

**Example** **A5.** 
*With λ=0.5,*

$$0.5\cdot A^{\left(1\right)}=\left[\begin{array}{cc} 0.3 & 0.15\\ 0.2 & 0.35 \end{array}\right]$$

**Example** **A6.** 
*Let α=0.5, then*

$${\left(A^{\left(1\right)}\right)}_{0.5}=\left[\begin{array}{cc} 1 & 0\\ 0 & 1 \end{array}\right]$$

**Example** **A7.** 

$$A^{\left(1\right)}\cup B^{\left(1\right)}=\left[\begin{array}{cc} \max(0.6,0.5) & \max(0.3,0.6)\\ \max(0.4,0.3) & \max(0.7,0.5) \end{array}\right]=\left[\begin{array}{cc} 0.6 & 0.6\\ 0.4 & 0.7 \end{array}\right]$$

$$A^{\left(1\right)}\cap B^{\left(1\right)}=\left[\begin{array}{cc} 0.5 & 0.3\\ 0.3 & 0.5 \end{array}\right]$$

**Example** **A8.** 

$$A^{\left(1\right)}=\left[\begin{array}{cc} 0.2 & 0.5\\ 0.9 & 0.6 \end{array}\right],\;A^{\left(2\right)}=\left[\begin{array}{cc} 0.1 & 0.4\\ 0.3 & 0.8 \end{array}\right]$$

*All entries lie within [0,1], so 𝒫 is bounded.*

**Example** **A9.** 

$$A^{\left(1\right)}=\left[\begin{array}{cc} 0.2 & 0.4\\ 0.3 & 0.5 \end{array}\right],\;B^{\left(1\right)}=\left[\begin{array}{cc} 0.3 & 0.5\\ 0.4 & 0.6 \end{array}\right]$$

*Since $A^{\left(1\right)}\leq B^{\left(1\right)}$ element-wise, we have*

ArithmeticMean:F(A)≤F(B)

**Example** **A10.** 

$$A^{\left(1\right)}=\left[\begin{array}{cc} 0.5 & 0.6\\ 0.7 & 0.8 \end{array}\right]\Rightarrow A^{\left(1\right)}\cup A^{\left(1\right)}=A^{\left(1\right)}$$

**Example** **A11.** 
*The same as in idempotency, any comparison with itself holds reflexively.*

**Example** **A12.** 

$$A^{\left(1\right)}=\left[\begin{array}{cc} 0.3 & 0.6\\ 0.8 & 0.4 \end{array}\right],\;B^{\left(1\right)}=\left[\begin{array}{cc} 0.7 & 0.4\\ 0.2 & 0.6 \end{array}\right],\;\lambda =0.5$$

$$C^{\left(1\right)}=0.5A^{\left(1\right)}+0.5B^{\left(1\right)}=\left[\begin{array}{cc} 0.5 & 0.5\\ 0.5 & 0.5 \end{array}\right]$$

**Example** **A13.** 

$$A^{\left(1\right)}=\left[\begin{array}{cc} 0.3 & 0.4 \end{array}\right],\;B^{\left(1\right)}=\left[\begin{array}{cc} 0.6 & 0.2 \end{array}\right],\;C^{\left(1\right)}=\left[\begin{array}{cc} 0.5 & 0.7 \end{array}\right]$$

$$\left(A^{\left(1\right)}\cup B^{\left(1\right)}\right)\cup C^{\left(1\right)}=\left[\begin{array}{cc} \max(0.3,0.6,0.5) & \max(0.4,0.2,0.7) \end{array}\right]=\left[\begin{array}{cc} 0.6 & 0.7 \end{array}\right]$$

**Example** **A14.** 

$$A^{\left(1\right)}=\left[0.4\right],\;B^{\left(1\right)}=\left[0.6\right]\Rightarrow A^{\left(1\right)}\cup B^{\left(1\right)}=B^{\left(1\right)}\cup A^{\left(1\right)}=\left[0.6\right]$$

**Example** **A15.** 
*Let*

$$A^{\left(1\right)}=\left[\begin{array}{cc} 0.4 & 0.6\\ 0.2 & 0.8 \end{array}\right],\;A^{\left(2\right)}=\left[\begin{array}{cc} 0.5 & 0.7\\ 0.3 & 0.6 \end{array}\right]$$

*Using the arithmetic mean,*

$$𝒯=\frac{1}{2}\left(A^{\left(1\right)}+A^{\left(2\right)}\right)=\left[\begin{array}{cc} 0.45 & 0.65\\ 0.25 & 0.7 \end{array}\right]$$

*All entries remain in [0,1], so 𝒯 is a PFT.*

**Example** **A16.** 
*Let*

$$A=\left[\begin{array}{cc} 0.3 & 0.6\\ 0.9 & 0.1 \end{array}\right],\;𝒫=\left\{A,A,A\right\}$$

*Then,*

max(𝒫)=min(𝒫)=A

**Example** **A17.** 

$$A=\left[\begin{array}{cc} 0.6 & 0.4\\ 0.7 & 0.9 \end{array}\right],\;\lambda =0.5\Rightarrow \lambda A=\left[\begin{array}{cc} 0.3 & 0.2\\ 0.35 & 0.45 \end{array}\right]$$

**Example** **A18.** 

$$A=\left[\begin{array}{cc} 0.2 & 0.5\\ 0.3 & 0.9 \end{array}\right]\Rightarrow A\cup A=A,\;A\cap A=A$$

**Example** **A19.** 

$$A=\left[\begin{array}{cc} 0.1 & 0.9\\ 0.3 & 0.7 \end{array}\right],\;B=\left[\begin{array}{cc} 0.6 & 0.4\\ 0.2 & 0.5 \end{array}\right],\;\lambda =0.4$$

$$C=0.4\cdot A+0.6\cdot B=\left[\begin{array}{cc} 0.4 & 0.6\\ 0.24 & 0.58 \end{array}\right]$$

**Example** **A20.** 
*Let fuzzy tensors A and B be given as*

$$A=\left[\begin{array}{cc} 0.2 & 0.7\\ 0.4 & 0.6 \end{array}\right],\;B=\left[\begin{array}{cc} 0.5 & 0.3\\ 0.8 & 0.2 \end{array}\right]$$

***Step 1: Union and Intersection***

$$A\cup B=\left[\begin{array}{cc} \max(0.2,0.5) & \max(0.7,0.3)\\ \max(0.4,0.8) & \max(0.6,0.2) \end{array}\right]=\left[\begin{array}{cc} 0.5 & 0.7\\ 0.8 & 0.6 \end{array}\right]$$

$$A\cap B=\left[\begin{array}{cc} \min(0.2,0.5) & \min(0.7,0.3)\\ \min(0.4,0.8) & \min(0.6,0.2) \end{array}\right]=\left[\begin{array}{cc} 0.2 & 0.3\\ 0.4 & 0.2 \end{array}\right]$$

***Step 2: Complements***

$$A^{c}=\left[\begin{array}{cc} 0.8 & 0.3\\ 0.6 & 0.4 \end{array}\right],\;B^{c}=\left[\begin{array}{cc} 0.5 & 0.7\\ 0.2 & 0.8 \end{array}\right]$$

$${\left(A\cup B\right)}^{c}=\left[\begin{array}{cc} 1-0.5 & 1-0.7\\ 1-0.8 & 1-0.6 \end{array}\right]=\left[\begin{array}{cc} 0.5 & 0.3\\ 0.2 & 0.4 \end{array}\right]$$

$$A^{c}\cap B^{c}=\left[\begin{array}{cc} \min(0.8,0.5) & \min(0.3,0.7)\\ \min(0.6,0.2) & \min(0.4,0.8) \end{array}\right]=\left[\begin{array}{cc} 0.5 & 0.3\\ 0.2 & 0.4 \end{array}\right]$$

$$\Rightarrow {\left(A\cup B\right)}^{c}=A^{c}\cap B^{c}$$

*Similarly:*

$${\left(A\cap B\right)}^{c}=\left[\begin{array}{cc} 0.8 & 0.7\\ 0.6 & 0.8 \end{array}\right],\;A^{c}\cup B^{c}=\left[\begin{array}{cc} \max(0.8,0.5) & \max(0.3,0.7)\\ \max(0.6,0.2) & \max(0.4,0.8) \end{array}\right]=\left[\begin{array}{cc} 0.8 & 0.7\\ 0.6 & 0.8 \end{array}\right]$$

$$\Rightarrow {\left(A\cap B\right)}^{c}=A^{c}\cup B^{c}$$

**Example** **A21.** 
*Let 𝒫 be a polytopic fuzzy tensor of order 2 with*

$$A^{\left(1\right)}=\left[\begin{array}{cc} 0.6 & 0.8\\ 0.7 & 0.5 \end{array}\right],\;A^{\left(2\right)}=\left[\begin{array}{cc} 0.4 & 0.9\\ 0.5 & 0.6 \end{array}\right]$$

*Then the aggregated tensor $A^{*}$ using arithmetic mean is*

$$A^{*}=\frac{1}{2}\left(\left[\begin{array}{cc} 0.6 & 0.8\\ 0.7 & 0.5 \end{array}\right]+\left[\begin{array}{cc} 0.4 & 0.9\\ 0.5 & 0.6 \end{array}\right]\right)=\left[\begin{array}{cc} 0.5 & 0.85\\ 0.6 & 0.55 \end{array}\right]$$

**Example** **A22.** 
*Using the previous tensors with weights $w_{1}=0.6$, $w_{2}=0.4$, we get*

$$A^{*}=0.6\cdot \left[\begin{array}{cc} 0.6 & 0.8\\ 0.7 & 0.5 \end{array}\right]+0.4\cdot \left[\begin{array}{cc} 0.4 & 0.9\\ 0.5 & 0.6 \end{array}\right]$$

$$A^{*}=\left[\begin{array}{cc} 0.36 & 0.48\\ 0.42 & 0.30 \end{array}\right]+\left[\begin{array}{cc} 0.16 & 0.36\\ 0.20 & 0.24 \end{array}\right]=\left[\begin{array}{cc} 0.52 & 0.84\\ 0.62 & 0.54 \end{array}\right]$$

**Example** **A23.** 
*Using the same matrices, we have the following.*

*Max-aggregation:*

$$A^{*}=\left[\begin{array}{cc} \max(0.6,0.4) & \max(0.8,0.9)\\ \max(0.7,0.5) & \max(0.5,0.6) \end{array}\right]=\left[\begin{array}{cc} 0.6 & 0.9\\ 0.7 & 0.6 \end{array}\right]$$

*Min-aggregation:*

$$A^{*}=\left[\begin{array}{cc} 0.4 & 0.8\\ 0.5 & 0.5 \end{array}\right]$$

**Example** **A24.** 
*Consider the same polytopic fuzzy tensor $𝒫=\{A^{\left(1\right)},A^{\left(2\right)}\}$ from Example A22. To aggregate the two tensors using the ordered weighted averaging (OWA) operator, we proceed element-wise. For each position $\left(j_{1},j_{2}\right)$, we order the values from the two tensors in descending order and apply a predefined weight vector $v={\left(0.7,0.3\right)}^{⊤}$ (summing to 1).*

*For instance, take element (1,1): the values are {0.6,0.4}. Sorting descending gives $b_{1}=0.6$, $b_{2}=0.4$. Applying OWA,*

$$A_{11}^{*}=0.7\cdot 0.6+0.3\cdot 0.4=0.42+0.12=0.54.$$

*Similarly, for element (1,2): values {0.8,0.9} become $b_{1}=0.9$, $b_{2}=0.8$:*

$$A_{12}^{*}=0.7\cdot 0.9+0.3\cdot 0.8=0.63+0.24=0.87.$$

*Repeating this procedure systematically for all elements yields the fully aggregated tensor $A^{*}$:*

$$A^{*}=\left[\begin{array}{cc} 0.54 & 0.87\\ 0.62 & 0.55 \end{array}\right].$$

*The complete element-wise calculation confirms the consistency of the OWA operator within the polytopic fuzzy tensor framework.*
